# Coronavirus Nsp3 Hijacks CLTC to Modulate Autophagosome Nucleation for Promoting DMV Formation and Viral Replication

**DOI:** 10.1002/advs.202521626

**Published:** 2026-02-21

**Authors:** Juan Xu, Hang Li, Peng Liu, Zhe Jiao, Ding Zhang, Zhelin Su, Sai Niu, Jintao Zhang, Yuejun Shi, Guiqing Peng

**Affiliations:** ^1^ State Key Laboratory of Agricultural Microbiology College of Veterinary Medicine Huazhong Agricultural University Wuhan P. R. China; ^2^ Key Laboratory of Preventive Veterinary Medicine in Hubei Province The Cooperative Innovation Center for Sustainable Pig Production Wuhan P. R. China; ^3^ State Key Laboratory of Agricultural Microbiology Key Laboratory of Prevention & Control for African Swine Fever and Other Major Pig Diseases Ministry of Agriculture and Rural Affairs College of Veterinary Medicine Huazhong Agricultural University Wuhan China

**Keywords:** autophagy, coronavirus, clathrin heavy chain, double‐membrane vesicle, live‐cell imaging, nonstructural protein 3

## Abstract

Double‐membrane vesicles (DMVs) are a hallmark of coronavirus replication, yet the host machinery governing their biogenesis remains poorly characterized, largely due to lack of tools for dynamic analysis. Here, we develop a live‐cell imaging system using a recombinant virus that enables, for the first time, real‐time visualization of DMV formation during authentic coronavirus infection. This system reveals the recruitment of the clathrin heavy chain (CLTC) to DMV assembly sites and demonstrates its essential role in diverse coronaviruses, but not in unrelated viruses. Notably, we define a previously unappreciated role of CLTC in viral replication organelle formation. Mechanistically, CLTC interacts with nonstructural protein 3 (nsp3) and is required for autophagosome nucleation by maintaining the core class III PI3K complex. The resulting CLTC‐mediated autophagic precursor membranes are subsequently hijacked by nsp3 to form DMVs. Our study thereby establishes CLTC as a pivotal host factor for coronavirus replication and nominates both CLTC and the autophagosome nucleation pathway as promising antiviral targets.

## Graphical abstract

In wild‐type cells, FIPV infection recruits CLTC to nsp3, facilitates DMV biogenesis and block autophagic flux to promote viral replication. CLTC knockout impairs autophagosome nucleation by reducing Beclin1–ATG14 complex expression. This disrupts the formation of autophagic precursor membranes, thereby preventing their hijacking by nsp3 for DMV assembly. Partial restoration of autophagic flux severely restricts DMV biogenesis and viral replication.

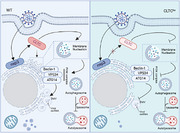

## 1 Introduction

Coronaviruses are single‐stranded positive‐sense RNA viruses with a broad host range, including humans, wildlife, companion animals, and livestock [1, 2, 3, 4, 5, 6]. Their zoonotic potential represents a significant public health threat, as evidenced by the viruses that caused two major epidemics—severe acute respiratory syndrome coronavirus (SARS‐CoV) and Middle East respiratory syrndrome coronavirus (MERS‐CoV)—and the unprecedented Coronavirus Disease 2019 (COVID‐19) pandemic caused by severe acute respiratory syndrome coronavirus 2 (SARS‐CoV‐2) [7, 8, 9]. More recently, a large‐scale outbreak of feline infectious peritonitis (FIP) caused by a highly pathogenic recombinant feline coronavirus, FCoV‐23, highlighted the inherent capacity of coronaviruses to undergo recombination, facilitating continuous cross‐species transmission risk [10, 11, 12].

Viruses adeptly manipulate host cellular machinery to enhance their replication. One notable mechanism involves the induction of extensive host membrane remodeling to support viral genome amplification. Positive‐strand RNA viruses, including coronaviruses, flaviviruses, picornaviruses, arteriviruses, and noroviruses, form conserved replication organelles known as double‐membrane vesicles (DMVs) [13, 14, 15]. These vesicles, which are typically 100–300 nm in diameter, consist of two tightly apposed membranes and often maintain connections with the endoplasmic reticulum (ER), suggesting that ER‐derived membranes contribute to their biogenesis, especially during early infection [16, 17]. Although morphologically similar to autophagosomes, the role of DMVs is distinct. Rather than mediating degradation, virus‐induced DMVs create a protective microenvironment that shields viral replication complexes from innate immune detection [18, 19, 20]. This compartmentalization is critical for efficient viral replication and immune evasion.

Viral nonstructural proteins (nsps) play a central role in the biogenesis of DMVs. With respect to SARS‐CoV‐2 and murine hepatitis virus (MHV), the co‐expression of nsp3 and nsp4 constitutes the minimal set of viral components required for DMV formation [21, 22]. This mechanism is conserved across coronaviruses: nsp3 and nsp4 of both SARS‐CoV and MERS‐CoV are similarly sufficient to induce DMV biogenesis, a phenomenon also observed in transmissible gastroenteritis virus (TGEV) [23, 24, 25]. Beyond coronaviruses, the induction of DMVs relies on distinct viral proteins across families: nsp2 and nsp3 in arteriviruses (e.g., equine arteritis virus), proteins 2BC and 3A in poliovirus, and the NS3–NS5B polyprotein in hepatitis C virus (HCV) [26, 27, 28]. Similarly, noroviruses require NS1‐2 and NS4 for DMV biogenesis [29].

During the formation of DMVs induced by viral proteins, multiple host factors are critically involved, with several of these factors functionally linked to the cellular autophagy pathway. Growing evidence indicates that coronaviruses exploit multiple components of the host autophagy machinery to facilitate DMV formation and maturation. For instance, the ER‐resident proteins Vacuole Membrane Protein 1 (VMP1) and Transmembrane Protein 41B (TMEM41B)—both essential for autophagosome formation—are utilized to induce membrane curvature and lipid remodeling during DMV biogenesis [30]. TMEM41B promotes the interaction between viral nonstructural proteins nsp3 and nsp4, while VMP1 modulates phospholipid composition, thereby ensuring DMV structural integrity and function [31]. Furthermore, the RNA‐binding proteins FMRP and FXR1/2, which undergo liquid–liquid phase separation to cluster viral replication organelles, share functional overlap with autophagy pathways, suggesting that autophagy‐related membranous compartments or their communication are essential for coronavirus‐induced DMVs [32, 33]. Additional autophagy‐linked host factors contribute critically to DMV formation. Acylglycerolphosphate acyltransferase (AGPAT) enzymes, which are involved in phospholipid biosynthesis and autophagosomal membrane expansion, support DMV development by providing essential lipid precursors [34]. Neutral sphingomyelinase 2, a regulator of autophagy flux and lipid raft organization, also influences DMV stability and viral replication efficiency [35]. Similarly, reticulon proteins RTN3 and RTN4, known to shape ER tubulation and participate in autophagosome formation, facilitate the membrane curvature required for DMV genesis [36]. In summary, coronaviruses selectively hijack autophagy‐related host factors not only to construct DMVs but also to subvert autophagic degradation. This strategic co‐opting of cellular membrane remodeling systems allows the virus to establish a protected replication niche, highlighting the intricate interplay between autophagy and DMV biogenesis during infection.

Despite significant advances, the molecular mechanisms and host cellular machinery governing DMV biogenesis remain incompletely understood. Previous studies have primarily relied on static imaging techniques such as transmission electron microscopy (TEM), which, while informative, cannot capture the dynamic process of DMV formation [37]. A major obstacle in the field has been the inability to visualize DMV dynamics in real time and to specifically isolate host factors associated with these transient viral structures. To overcome this limitation, we sought to develop a versatile tool for the simultaneous live imaging and proteomic profiling of DMV‐associated host proteins. We hypothesized that a recombinant virus expressing a fluorescently tagged version of a core viral scaffolding protein could achieve this dual objective.

Coronavirus nsp3 is recognized as one of the minimal essential components for the formation of the replication organelle [19, 22]. Although nsp3 exhibits variations in domain organization across different coronavirus genera (e.g., α‐ versus β‐coronaviruses), its core functions, such as serving as a scaffold for the replication‐transcription complex (RTC), facilitating membrane rearrangement, and interacting with other viral non‐structural proteins (e.g., nsp4 and nsp6), are remarkably conserved across the virus family [38, 39]. This functional conservation suggests that insights gained from one coronavirus system can inform mechanisms in others. To investigate the conserved process of DMV biogenesis, we selected the nsp3 of feline infectious peritonitis virus (FIPV)—an α‐coronavirus with demonstrated cross‐species potential—for fluorescent tagging [10, 22]. Studies in this model system provide valuable mechanistic insights relevant to β‐coronaviruses such as SARS‐CoV‐2.

Here, we provide a powerful new approach for studying viral replication organelles and reveal the previously unrecognized role of clathrin heavy chain (CLTC) mediated autophagy nucleation in the life cycle of coronaviruses, highlighting potential therapeutic targets for antiviral intervention.

## 2 Results

### 2.1 Construction of a Novel Recombinant FIPV Nsp3‐ZsGreen

To enable real‐time visualization of DMVs and facilitate the identification of host factors involved in their biogenesis, we engineered a recombinant FIPV expressing a ZsGreen fluorescent protein fused to nsp3. ZsGreen was selected because of its strong luminescence and rapid maturation, making it particularly suitable for dynamic live‐cell imaging of viral replication processes [40].

**FIGURE 1:**
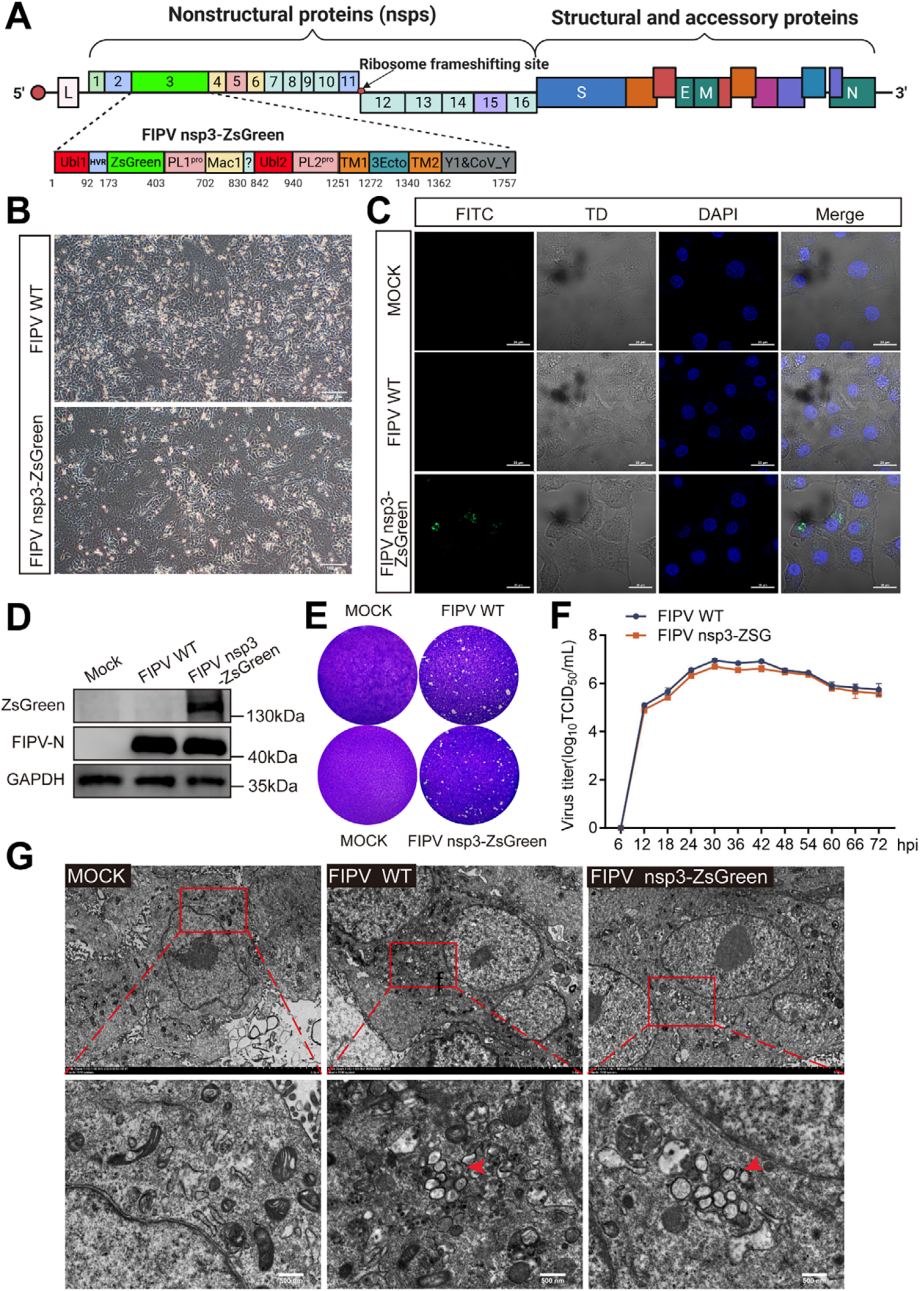
Construction of a novel recombinant FIPV nsp3‐ZsGreen. (A) Schematic representation of the recombinant FIPV nsp3‐ZsGreen virus construct. The hypervariable region (residues 173–180) is replaced with a ZsGreen fluorescent protein tag (highlighted in green), enabling visualization of nsp3 localization. Created with BioRender.com. https://BioRender.com/v7qqf6l (B) Comparative cytopathic effect (CPE) analysis of rescued FIPV nsp3‐ZsGreen and FIPV WT in CRFK cells. Scale bar = 100 µm. (C) Immunofluorescence analysis of CRFK cells infected with FIPV nsp3‐ZsGreen or FIPV WT (MOI = 0.1, 18 hpi). ZsGreen fluorescence (green) localizes to cytoplasmic foci during viral replication, with DAPI nuclear staining (blue) confirming cell viability. Scale bar = 20 µm. (D) Western blot analysis of the expression of the ZsGreen‐tagged fusion protein in CRFK cells infected with FIPV nsp3‐ZsGreen and FIPV WT (MOI = 1, 12 hpi). (E) Comparative plaque assay between FIPV nsp3‐ZsGreen and FIPV WT (MOI = 0.1, 24 hpi) in CRFK cells stained with crystal violet. (F) Multistep growth curves of FIPV nsp3‐ZsGreen and FIPV WT in CRFK cell (MOI = 0.1). The infectious virus titer in CRFK cells sampled at the indicated time points was determined by end‐point dilution and calculated as TCID_50_/mL. Data represent the means ± SD from three independent experiments. Statistical significance was determined using a two‐tailed Student's *t*‐test. (G) TEM analysis of DMV biogenesis in CRFK cells infected with FIPV nsp3‐ZsGreen or FIPV WT (MOI = 5, 9 hpi). Representative micrographs show DMVs (arrows) localized within the cytoplasm. Scale bars = 500 nm (main panels) and 100 nm (insets).

To identify an appropriate site for ZsGreen insertion within nsp3, we conducted a sequence alignment of nsp3 from FIPV QS‐79 (FCoV type I) and FIPV 79–1146 (FCoV type II). This comparison revealed an 8‐amino acid insertion (residues 173–180) within a hypervariable region of FIPV 79–1146. Consistent with previous reports that nsp3 hypervariable regions often form surface‐exposed loops, structural prediction indicated that this site is located within a flexible loop [39]. We therefore replaced residues 173–180 with the ZsGreen sequence (Figure 1A and Figure S1A). The recombinant virus, designated FIPV nsp3‐ZsGreen, was successfully rescued using a bacterial artificial chromosome (BAC)‐based reverse genetics system. Rescue was confirmed by the appearance of characteristic cytopathic effects and robust green fluorescence in infected cultures (Figure 1B,C). Western blot analysis of lysates from infected feline kidney (CRFK) cells detected a single band of approximately 190 kDa, which corresponds to the predicted molecular weight of the nsp3‐ZsGreen fusion protein and is considerably larger than the 26 kDa band of ZsGreen alone. These results were further supported by fluorescence size‐exclusion chromatography (FSEC; Figure 1D and Figure S1B). The genetic stability of the reporter virus was assessed by serial passage in CRFK cells over 20 generations (P1–P20). Examination at every fifth passage via fluorescence microscopy, PCR, and Sanger sequencing confirmed that the ZsGreen insertion remained intact throughout (Figure S1C–E), supporting the robustness of the recombinant construct.

To characterize the replication kinetics of the recombinant virus FIPV nsp3‐ZsGreen in CRFK cells, we performed plaque assays and generated multistep growth curves for comparison with those of FIPV 79–1146 (FIPV WT). These results (Figure 1E,F) revealed that the replication kinetics of the two viruses were similar in CRFK cells. Furthermore, TEM confirmed that infection with FIPV nsp3‐ZsGreen induced the formation of DMVs that were indistinguishable from those produced during FIPV WT infection (Figure 1G).

### 2.2 FIPV Nsp3‐ZsGreen Enables Real‐Time Visualization of DMV Biogenesis During Coronavirus Infection

**FIGURE 2:**
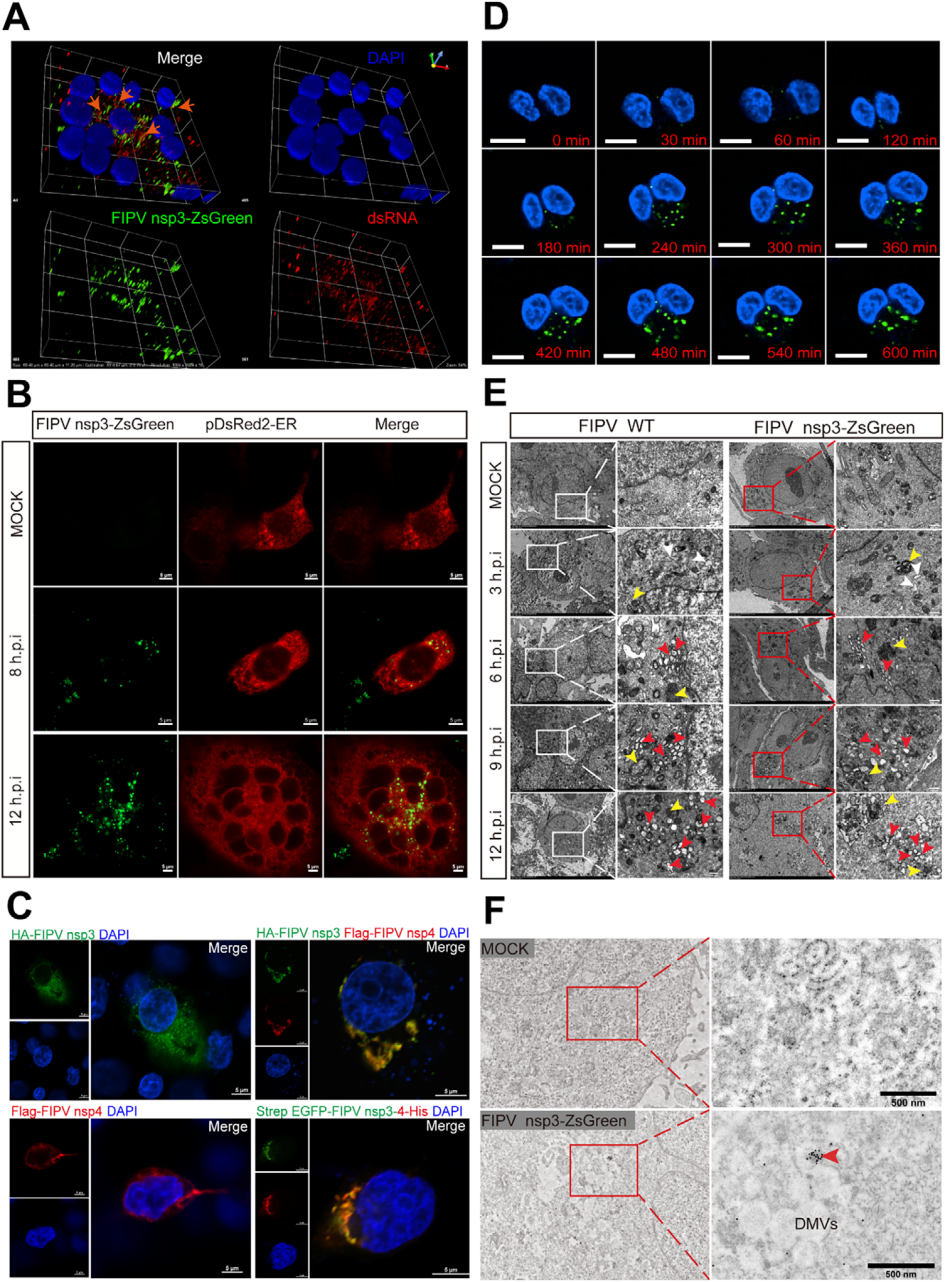
FIPV nsp3‐ZsGreen as a novel tool for real‐time imaging of DMV biogenesis during coronavirus infection. (A) Three‐dimensional confocal microscopy analysis of dsRNA (red) and ZsGreen‐labeled DMVs (green) in CRFK cells infected with FIPV nsp3‐ZsGreen (MOI = 5, 9 hpi). Maximum intensity projections reconstructed from 21 optical sections (100× magnification) showing colocalization of DMVs with dsRNA (arrowheads). Scale bar = 5 µm. The images were processed using NIS‐Elements software. (B) Live‐cell imaging of FIPV nsp3‐ZsGreen colocalization with the endoplasmic reticulum. CRFK cells were transfected with the pDsRed‐ER plasmid (red, ER marker) for 18 h and subsequently infected with FIPV nsp3‐ZsGreen (green, MOI = 5) for 8 h and 12 h. Scale bar = 5 µm. (C) CRFK cells were transfected with HA‐FIPV nsp3 (upper left), Flag‐FIPV nsp4 (lower left) or Strep EGFP‐FIPV nsp3‐4‐His (lower right) or co‐transfected with HA‐FIPV nsp3 and Flag‐FIPV nsp4 (upper right) for 48 h. Cells were fixed and stained with anti‐HA (green), anti‐Flag (red), anti‐Strep (green), anti‐His (red), and DAPI (blue). Scale bar = 5 µm. (D) Real‐time imaging of CRFK cells infected with FIPV nsp3‐ZsGreen (MOI = 10) from 4 hpi to 18 hpi. Scale bar = 10 µm. (E) CRFK cells were infected with FIPV WT or FIPV nsp3‐ZsGreen (MOI = 5) and processed for transmission electron microscopy analysis at 3, 6, 9, 12 hpi. The areas in the white (FIPV WT) and red (FIPV nsp3‐ZsGreen) boxes are shown in the corresponding EM images. Arrowheads indicate DMVs (red), autophagosomes (yellow) and ER (white). Scale bar = 500 nm. (F) The indicated cells were mock inoculated or infected with FIPV nsp3‐ZsGreen (MOI = 5, 9 hpi) and processed for immunogold EM. Labeled FIPV nsp3‐ZsGreen is indicated with an arrowhead. Scale bar = 500 nm.

Previous studies have shown that coronavirus nsp3 contributes to the formation of DMVs [13] and that double‐stranded RNA (dsRNA) serves as a reliable marker of these structures [41]. To evaluate whether FIPV nsp3‐ZsGreen can accurately track DMV dynamics, we examined infected CRFK cells using confocal microscopy. Results revealed pronounced spatial colocalization between nsp3‐ZsGreen and dsRNA (Figure 2A). Given the ER origin of DMVs, we also detected the colocalization of nsp3‐ZsGreen with endoplasmic reticulum markers (Figure 2B).

It has been previously demonstrated that the coexpression of coronavirus nsp3 and nsp4 is sufficient to induce DMV formation [22]. Consistent with these reports, expression of either nsp3 or nsp4 alone led to diffuse cytoplasmic localization. In contrast, coexpression of nsp3 and nsp4—or expression of an nsp3–4 fusion construct—resulted in the formation of distinct perinuclear puncta (Figure 2C). Notably, a perinuclear punctate fluorescent pattern was observed in cells infected with FIPV nsp3‐ZsGreen. To rule out this pattern as a nonspecific artifact of the ZsGreen tag, we constructed a control virus (FIPV deORF3‐ZsGreen) in which ZsGreen replaced the open reading frame (ORF) 3 gene. This control virus exhibited replication kinetics comparable to those of both FIPV nsp3‐ZsGreen and FIPV WT. However, cells infected with FIPV deORF3‐ZsGreen displayed diffuse cytoplasmic fluorescence, a pattern clearly distinct from the punctate pattern associated with the nsp3‐ZsGreen fusion protein (Figure S2A, C). Together, these findings support the association of nsp3‐ZsGreen with DMVs.

Live‐cell imaging of CRFK cells infected with FIPV nsp3‐ZsGreen revealed that fluorescence signals were initially detectable at 3 hours post‐infection (hpi). Both the intensity and the number of fluorescent puncta increased progressively over time (Figure S2B). Furthermore, long‐term live‐cell imaging of infected cells revealed that fluorescent signals emerged during the early phase of infection and progressively formed punctate foci in the perinuclear region over time (Figure 2D). TEM further confirmed that infection with FIPV nsp3‐ZsGreen induced DMVs that were morphologically comparable to those observed during FIPV WT infection, with the number of DMVs increasing throughout infection (Figure 2E). These results align with previously established timelines of DMV biogenesis.

Subsequent immunoelectron microscopy (IEM) analysis revealed specific immunogold labeling of ZsGreen on DMV membranes in FIPV nsp3‐ZsGreen‐infected cells, which was consistent with the documented distribution of nsp3 (Figure 2F). Taken together, the colocalization with DMV markers, temporal expression dynamics, and ultrastructural evidence indicate that FIPV nsp3‐ZsGreen constitutes a novel and effective tool for the real‐time imaging of DMV biogenesis during coronavirus infection.

### 2.3 Identification of Host Factors Associated with FIPV‐Induced DMVs and Functional Validation of CLTC

**FIGURE 3:**
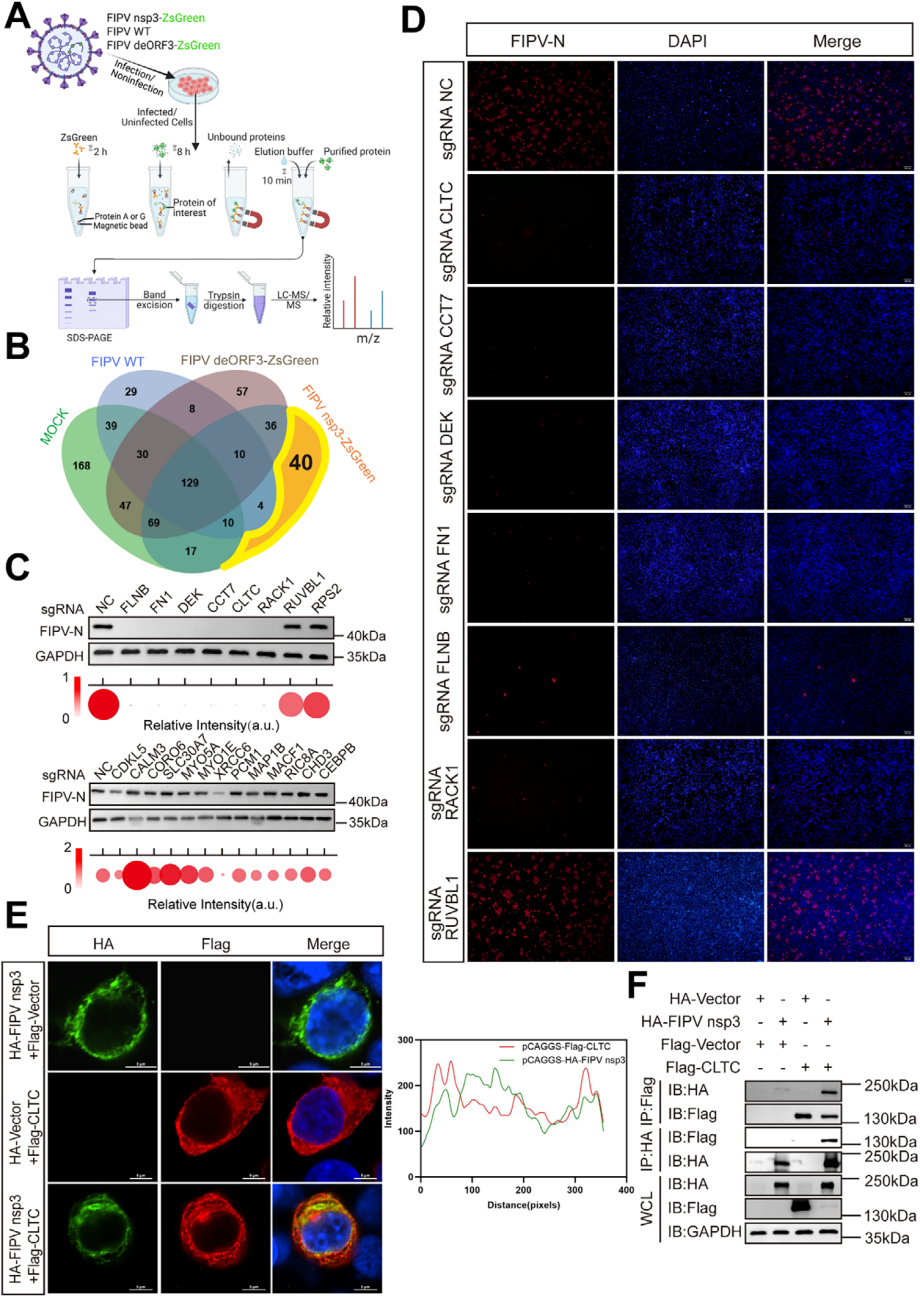
Screening and identification of critical host factors involved in DMV formation. (A) Flowchart showing the experimental design for identifying DMV‐related factors. Lysates from CRFK cells that were mock inoculated or infected with FIPV WT, FIPV deORF3‐ZsGreen, or FIPV nsp3‐ZsGreen (MOI = 5, 9 hpi) were subjected to ZsGreen‐Trap, followed by MS analysis. The first three groups served as controls. Created with BioRender.com. https://BioRender.com/kuepp5g (B) Venn diagram of the MS data of the four groups showing the 40 specific host factors identified from the FIPV nsp3‐ZsGreen group. Mock: mock inoculated; FIPV WT: infected with FIPV WT; FIPV deORF3‐ZsGreen: infected with FIPV deORF3‐ZsGreen; FIPV nsp3‐ZsGreen: infected with FIPV nsp3‐ZsGreen. (C, D) CRFK pool cells with knockout of the indicated DMV‐related host factors were infected with FIPV nsp3‐ZsGreen (MOI = 0.1, 12 hpi), NP protein expression levels were determined by western blot with GAPDH serving as a loading control (C). Cells were fixed and stained with anti‐NP (red) and DAPI (blue) for immunofluorescence analysis (D). Scale bar = 200 µm. (E, F) HEK293T cells were co‐transfected with pCAGGS‐HA FIPV nsp3 and pCAGGS‐Flag‐Vector, pCAGGS‐HA‐Vector and pCAGGS‐Flag‐CLTC, or pCAGGS‐HA FIPV nsp3 and pCAGGS‐Flag‐CLTC for 48 h. Cells were fixed and stained with anti‐HA (green), anti‐Flag (red) and DAPI (blue). Scale bar = 5 µm. The colocalization of pCAGGS‐HA FIPV nsp3 with pCAGGS‐Flag‐CLTC was analyzed using Fiji (E). Co‐IP was performed using anti‐Flag or anti‐HA antibodies (F).

To systematically identify host factors involved in the biogenesis of DMVs during FIPV infection, we employed an affinity purification mass spectrometry (AP‒MS) approach. A flowchart of the experimental design is shown (Figure 3A). Lysates from CRFK cells that were mock inoculated or infected with FIPV WT, FIPV deORF3‐ZsGreen, or FIPV nsp3‐ZsGreen (MOI = 5, 9 hpi) were subjected to ZsGreen‐Trap immunoprecipitation and subsequent mass spectrometric analysis. Comparative bioinformatics analysis of the four groups identified 40 host proteins that were specifically enriched in the FIPV nsp3‐ZsGreen‐infected group, suggesting their potential association with nsp3‐anchored DMVs (Figure 3B).

Of these candidates, we selected 21 for functional validation of their role in viral replication. Using the CRISPR/Cas9 system, we generated a series of CRFK pool cells with individual knockout of each candidate gene. Upon infection with FIPV nsp3‐ZsGreen (MOI = 0.1, 12 hpi), the CLTC, CCT7, DEK, FN1, FNLB, and RACK1 knockout pools showed substantial reductions in viral nucleocapsid protein (NP) levels, as determined by western blot (Figure 3C), along with a corresponding decrease in the number of NP‐positive cells, as determined by immunofluorescence analysis (Figure 3D), indicating that these factors are critical for efficient FIPV replication.

Among the top candidates, CLTC was selected for further mechanistic investigation. Cotransfection of HEK293T cells with plasmids expressing HA‐tagged FIPV nsp3 and Flag‐tagged CLTC revealed strong colocalization of the two proteins by confocal microscopy (Figure 3E). This interaction was further confirmed by coimmunoprecipitation (co‐IP) assays, which revealed that FIPV nsp3 specifically binds to CLTC (Figure 3F). In addition, similar subcellular localization and colocalization of nsp3 and CLTC were noted in FIPV nsp3‐ZsGreen‐infected cells (Figure S3A), confirming the strong association between nsp3 and CLTC. Finally, co‐IP experiments in cells transfected with HA‐nsp3 confirmed that nsp3 also interacted with endogenous CLTC (Figure S3B).

Collectively, these results identify a set of host factors specifically associated with FIPV nsp3‐induced DMVs and establish an interaction between viral nsp3 and the host membrane remodeling protein CLTC.

### 2.4 CLTC is a Critical Host Factor for Coronavirus Infection

**FIGURE 4:**
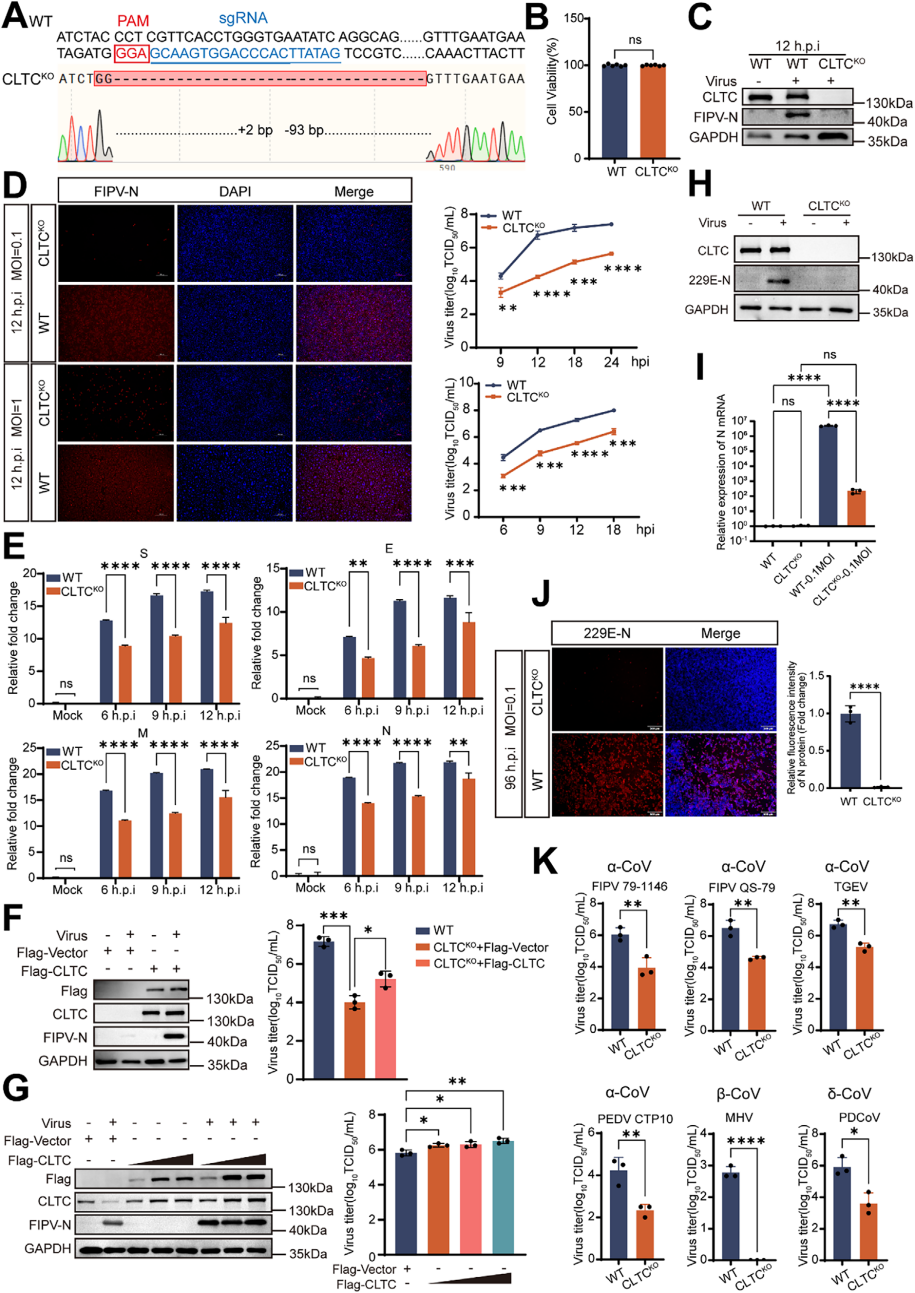
CLTC is a critical host factor for coronavirus infection. (A) Sanger sequencing of CLTC knockout cells. (B) Cell viability of WT and CLTC KO cells was determined by MTS assay (n = 6).  Data are mean ± SD (unpaired two‐tailed Student's *t*‐test). ns: p ≥ 0.05. (C) Western blot analysis of CLTC knockout efficiency and its effect on viral protein levels (FIPV nsp3‐ZsGreen, MOI = 0.1, 12 hpi). (D) WT and CLTC KO cells were infected with FIPV nsp3‐ZsGreen at an MOI of 0.1 or 1 for 12 h. Cells were fixed, permeabilized, and stained with an anti‐NP antibody (red) and DAPI (blue). Scale bar = 100 µm. The infected cell supernatants were subsequently harvested at the indicated times, and virus titers were determined by a TCID_50_ assay (n = 3). Data are mean ± SD (unpaired two‐tailed Student's *t*‐test). ^**^ *p* < 0.01; ^***^ *p* < 0.001; ^****^ *p* < 0.0001. (E) Time‐course measurement of relative RNA levels of S, E, N, M in WT and CLTC KO cells following FIPV nsp3‐ZsGreen (MOI = 1) infection (n = 3). Data are mean ± SD (unpaired two‐tailed Student's *t*‐test). ns: p ≥ 0.05; ^**^ *p* < 0.01; ^***^ *p* < 0.001; ^****^ *p* < 0.0001. (F) CLTC KO cells were transfected with 1 µg of exogenous CLTC or an empty vector as a negative control for 36 h, and then infected with FIPV nsp3‐ZsGreen (MOI = 1, 12 hpi). Flag, CLTC, and NP protein levels were determined by western blot, with GAPDH serving as a loading control. (G) CRFK cells were transfected with 0.5, 1, 1.5 µg of exogenous CLTC or an empty vector as a negative control for 36 h, and then infected with FIPV nsp3‐ZsGreen (MOI = 0.1, 12 hpi). Flag, CLTC and NP protein levels were determined by western blot, with GAPDH serving as a loading control. Viral titers in (F, G) supernatants were quantified using a TCID_50_ assay (n = 3) on CRFK cells. Data are mean ± SD (one‐way ANOVA with Dunnett's test against the respective control group). ^*^ *p* < 0.05; ^**^ *p* < 0.01; ^***^ *p* < 0.001. (H) Western blot analysis of CLTC expression confirms knockout efficiency in Huh‐7 cells and shows the impact on HCoV‐229E infection (MOI = 0.1, 48 hpi) in WT versus CLTC KO cells. (I) Relative expression levels of N protein in Huh‐7 WT and CLTC KO cells following HCoV‐229E (MOI = 0.1) infection (n = 3). Data are mean ± SD (two‐way ANOVA with Tukey's multiple comparisons test). ns: p ≥ 0.05; ^****^ *p* < 0.0001. (J) Huh‐7 WT and CLTC KO cells were infected with HCoV‐229E at an MOI of 0.1 for 96 h. Cells were fixed, permeabilized, and stained with an anti‐NP antibody (red) and DAPI (blue). Scale bar = 500 µm. The relative fluorescence intensity of NP staining was subsequently quantified (n = 3). Data are mean ± SD (unpaired two‐tailed Student's *t*‐test). ^****^ *p* < 0.0001. (K) Broad‐spectrum antiviral analysis. CRFK WT and CLTC KO cells were infected with α‐coronavirus: FIPV 79–1146, FIPV QS‐79, TGEV, PEDV CTP10; β‐coronavirus: MHV; δ‐coronavirus: PDCoV; all at MOI = 1, 12 hpi). Viral titers in the supernatants were quantified using a TCID_50_ assay (n = 3) on CRFK cells. Data are mean ± SD (unpaired two‐tailed Student's *t*‐test). ^*^ *p* < 0.05; ^**^ *p* < 0.01; ^****^ *p* < 0.0001.

To investigate the role of CLTC in FIPV infection, we generated CLTC‐knockout (CLTC KO) cells using the CRISPR/Cas9 system. Successful knockout was confirmed by genomic sequencing and western blot, with no significant effect on cell viability (Figure 4A–C). Infection with FIPV nsp3‐ZsGreen showed that CLTC knockout markedly reduced both intracellular viral nucleocapsid protein (NP) levels and viral titers (Figure 4D).

We next examined whether CLTC depletion affects viral structural protein expression. Quantitative RT‒PCR analysis revealed that viral RNA levels were reduced in CLTC KO cells throughout infection (Figure 4E), indicating suppressed viral gene expression. This effect was specifically attributable to loss of CLTC, as reintroduction of FLAG‐tagged CLTC into knockout cells restored NP expression and viral titers (Figure 4F). Conversely, overexpression of CLTC in wild‐type (WT) cells significantly increased both FIPV titers and intracellular NP accumulation (Figure 4G).

To further validate the relevance of our findings in a human system, we generated CLTC‐knockout human Huh‐7 cells (Figure 4H and Figure S4A, B). Upon infection with the human coronavirus HCoV‐229E, we observed a profound inhibition of viral replication, evidenced by significantly reduced viral RNA levels and nucleocapsid protein expression (Figure 4H–J). To determine whether CLTC dependency extends to other viruses, we evaluated a broad panel of viral pathogens. The replication of diverse coronaviruses—including FIPV strains 79–1146 and QS‐79, TGEV, porcine epidemic diarrhea virus strains CTP10 and YN200 (PEDV CTP10 and PEDV YN200), MHV, and porcine δ‐coronavirus (PDCoV)—was significantly impaired in CLTC‐knockout cells compared with WT controls. In contrast, the replication of vesicular stomatitis virus (VSV; a negative‐sense RNA virus) and the DNA viruses pseudorabies virus (PRV) and herpes simplex virus (HSV) remained unaffected (Figure 4K and Figure S4C). Together with our data on MHV (a β‐coronavirus), these findings establish that CLTC is a conserved host factor essential for both human and animal coronaviruses across different genera.

### 2.5 CLTC Knockout Impairs Virus Replication by Disrupting DMV Formation

**FIGURE 5:**
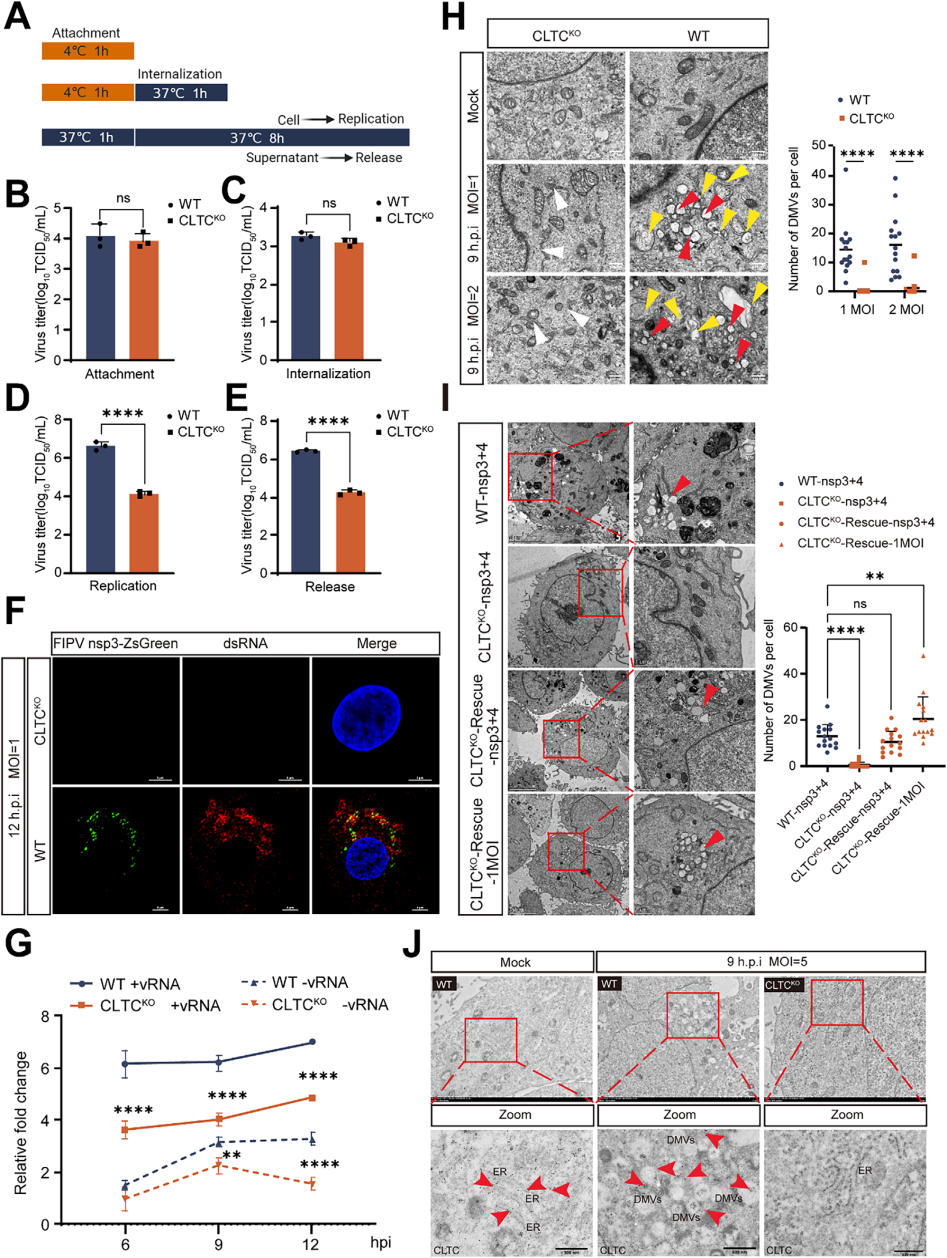
Knockout of CLTC inhibits viral replication through suppression of DMV formation. (A) Graphic representation of the designed viral infection stage assay in CRFK cells. (B, C) WT and CLTC KO cells were infected with FIPV nsp3‐ZsGreen at an MOI of 10 for attachment (B) or internalization (C)assays. (D, E) WT and CLTC KO cells were infected with FIPV nsp3‐ZsGreen (MOI = 1, 9 hpi) for replication (D) and release (E) assays. Viral titers at different infection stages were quantified using a TCID_50_ assay in CRFK cells (B‐E) (n = 3). Data are mean ± SD (unpaired two‐tailed Student's *t*‐test). ns: p ≥ 0.05; ^****^ *p* < 0.0001. (F) Colocalization of nsp3‐ZsGreen and dsRNA following FIPV nsp3‐ZsGreen (green) (MOI = 1, 12 hpi) infection in WT and CLTC KO cells. Immunofluorescence staining of dsRNA (red) and DAPI (blue) was performed. Scale bar = 5 µm. (G) WT and CLTC KO cells were infected with FIPV nsp3‐ZsGreen at an MOI of 1. Positive (+vRNA) or negative (−vRNA) viral RNA was quantified by quantitative real‐time PCR (n = 3). Data are mean ± SD (two‐way ANOVA with Tukey's multiple comparisons test). ^**^ *p* < 0.01; ^****^ *p* < 0.0001. (H) WT and CLTC KO cells were mock inoculated or infected with FIPV nsp3‐ZsGreen at an MOI of 1 or 2. Cells were fixed in glutaraldehyde at 9 hpi, and the formation of DMVs was observed using TEM. DMVs are indicated with red arrowheads, autophagosomes are indicated with yellow arrowheads, and the ER is indicated with white arrowheads. Quantitative analysis of the number of DMVs per cell across the indicated groups is shown(n = 15). Data are mean ± SD (unpaired two‐tailed Student's *t*‐test). ^****^ *p* < 0.0001. Scale bar = 500 nm. (I) Representative TEM images of with or without CLTC in CRFK cells under various conditions.  Images show WT or CLTC KO cells co‐transfected with nsp3 and nsp4 plasmids; CLTC KO cells transfected with a CLTC‐expressing plasmid prior to nsp3/nsp4 co‐transfection; and CLTC KO cells infected with FIPV (MOI = 1, 12 hpi). Quantitative analysis of the number of DMVs per cell across the indicated groups (n = 15). Data are mean ± SD (one‐way ANOVA with Dunnett's test against the respective control group). ns: p ≥ 0.05; ^**^ *p* < 0.01; ^****^ *p* < 0.0001. Scale bar = 5 µm and 1 µm (insets). (J) WT and CLTC KO cells were mock inoculated or infected with FIPV nsp3‐ZsGreen (MOI = 5, 9 hpi), after which immunogold labeling of the CLTC protein (red arrowhead) was performed for IEM detection. Scale bar = 500 nm.

To determine at which stage of the viral life cycle CLTC influences FIPV proliferation, we systematically evaluated the effects of CLTC knockout on the attachment, internalization, replication, and release of FIPV nsp3‐ZsGreen (Figure 5A). Using high multiplicity of infection (MOI = 10), we observed that CLTC deficiency did not significantly affect viral attachment to the cell surface or internalization (Figure 5B,C), indicating that early entry steps are CLTC‐independent.

In contrast, at a lower MOI (MOI = 1) to investigate events after entry, CLTC knockout markedly reduced intracellular viral RNA replication and the subsequent release of progeny virions (Figure 5D,E), suggesting that CLTC plays a critical role in viral genome amplification and/or particle production.

Since coronavirus replication is associated with the formation of DMVs, we investigated whether CLTC is involved in this process. Immunofluorescence analysis revealed a significant reduction in the colocalization of nsp3‐ZsGreen (a marker of viral replication complexes) and dsRNA (a replication intermediate) in CLTC KO cells compared with WT control cells (Figure 5F). Consistent with this disruption, CLTC knockout severely attenuated intracellular viral RNA synthesis (Figure 5G). Together, these data indicate that the formation of functional replication organelles is impaired.

To directly visualize the DMVs, we performed TEM on the infected cells. While numerous DMVs (indicated by red arrowheads) were evident in WT cells at 9 hpi, the formation of DMVs was markedly reduced in CLTC KO cells (Figure 5H). Quantitative analysis confirmed a significant decrease in the number of DMVs per cell in the knockout group. Notably, autophagosome‐like structures (yellow arrowheads) were still observable, indicating a specific defect in virus‐induced membrane remodeling rather than the general suppression of all double‐membrane cellular structures. To rigorously confirm that the defect in DMV formation was directly caused by CLTC deficiency rather than off‐target effects, we performed a TEM‐based structural rescue experiment. Utilizing the nsp3‐nsp4 co‐expression system to induce DMVs, we observed that DMV formation was abolished in CLTC KO cells. However, re‐expression of CLTC in these knockout cells successfully rescued the phenotype, restoring the number of DMVs to levels comparable to wild‐type cells (Figure 5I). This structural restoration confirms that CLTC is not only required but is a central functional component in the assembly of coronavirus replication organelles.

Furthermore, IEM demonstrated specific enrichment of CLTC protein (red arrowheads) near DMVs in infected WT cells (Figure 5J), providing spatial evidence that CLTC is recruited to viral replication organelles.

Collectively, these results demonstrate that CLTC is dispensable for FIPV entry but is essential for the formation of DMVs, which serve as sites of viral RNA synthesis, thereby facilitating efficient viral replication.

### 2.6 Spatiotemporal Dynamics and Molecular Mapping of the Nsp3‐CLTC Interaction

**FIGURE 6:**
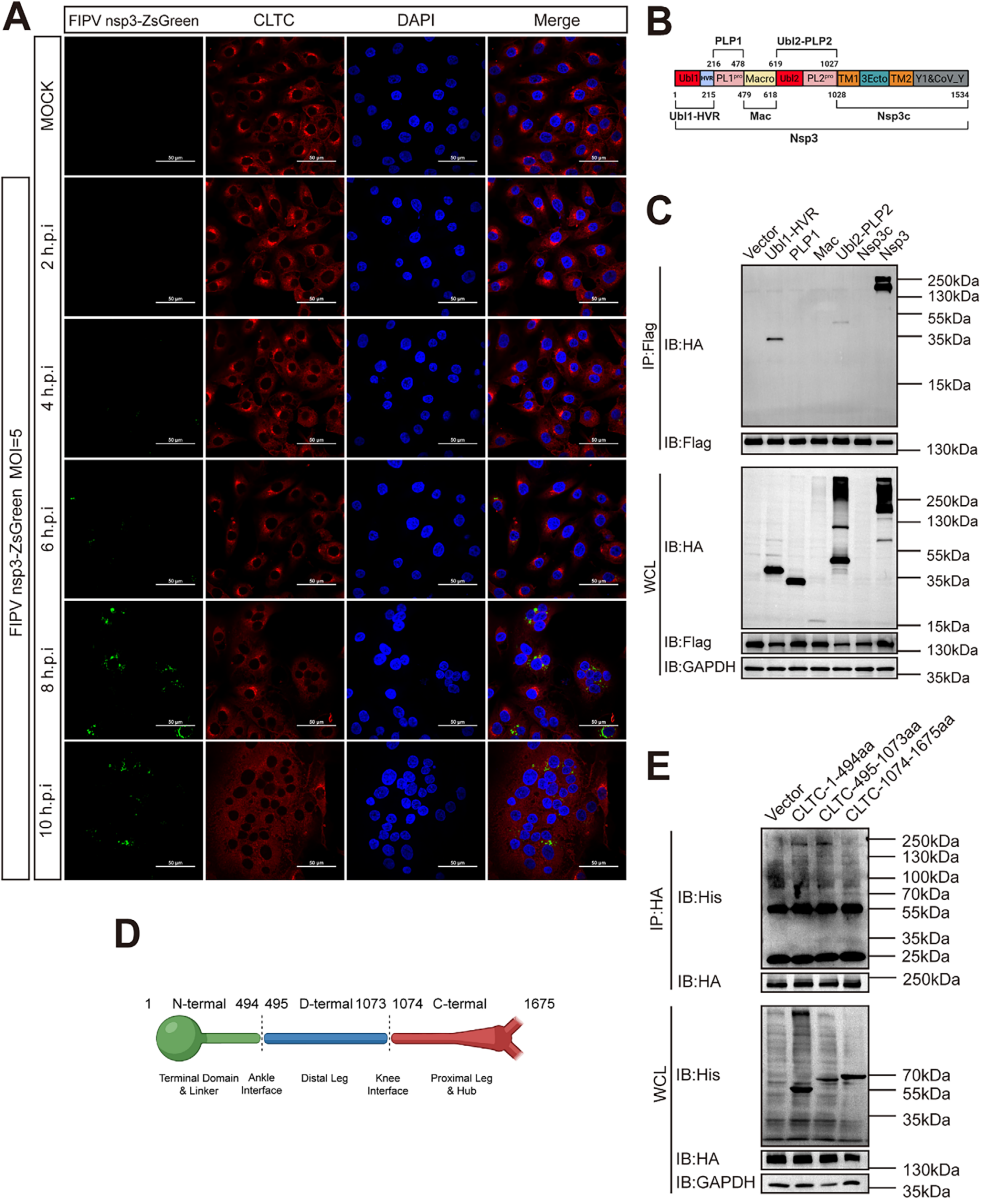
Spatiotemporal dynamics and molecular mapping of the FIPV nsp3‐CLTC interaction. (A) Temporal recruitment of endogenous CLTC to viral replication sites. CRFK cells were infected with FIPV nsp3‐ZsGreen (green) at an MOI of 5 and fixed at the indicated time points (2, 4, 6, 8, and 10 hpi). Endogenous CLTC (red) and nuclei (DAPI, blue) were visualized by immunofluorescence. Note the progressive transition of CLTC from a diffuse cytoplasmic distribution to distinct puncta that colocalize with nsp3‐ZsGreen, particularly at 8 and 10 hpi. Scale bar = 50 µm. (B) Schematic of the domain architecture of FIPV nsp3. Created with BioRender.com. https://BioRender.com/5b4wzoc (C) Mapping the CLTC‐binding domain within nsp3. HEK293T cells were co‐transfected with Flag‐tagged CLTC and the indicated HA‐tagged nsp3 truncation mutants for 36 h. Co‐immunoprecipitation was performed using anti‐Flag magnetic beads. Immunoblot analysis of the precipitates and whole‐cell lysates revealed that CLTC specifically interacts with the Ubl1‐HVR and Ubl2‐PLP2 domains. (D) Schematic representation of the functional domains of feline CLTC. The protein is segmented into the N‐terminal domain (1–494 aa), Distal Leg (495–1073 aa), and Proximal Leg & Hub (1074–1675 aa) for interaction mapping. (E) Analysis of the CLTC structural domains involved in the interaction. HEK293T cells were co‐transfected with HA‐tagged nsp3 and His‐tagged CLTC deletion mutants for 36 h. Lysates were subjected to immunoprecipitation using anti‐HA magnetic beads overnight. The interaction profile of the CLTC fragments was analyzed by immunoblotting with anti‐His antibody.

To define the dynamics of CLTC engagement with the viral replication machinery, we monitored the localization of endogenous CLTC in FIPV‐infected cells over time. We observed a time‐dependent rearrangement of CLTC from its conventional steady‐state distribution to nsp3‐ZsGreen‐positive puncta. By 8 and 10 hpi, CLTC localization was markedly enriched at these viral replication sites, demonstrating a concomitant recruitment with the peak formation of viral replication organelles (Figure 6A).

To define the precise viral determinants for CLTC binding, we performed a domain‐mapping analysis of FIPV nsp3 (Figure 6B). Co‐immunoprecipitation assays in HEK293T cells showed that CLTC specifically interacted with the Ubl1‐HVR and Ubl2‐PLP2 domains of nsp3, but not with other truncation mutants (Figure 6C). Sequence alignment analysis further demonstrated that these two domains share conserved structural motifs across multiple coronaviruses (Figure S5C). Taken together, the exceptional evolutionary conservation of CLTC across host species (>99.9% identity) and the modeled CLTC‐nsp3 interface provide a structural rationale for the broad requirement of CLTC across diverse coronaviruses(Figure S5A, B). The engagement of conserved viral Ubl2/PLP2 domains with an invariant host factor provides a structural explanation for the broad‐spectrum requirement of CLTC in coronavirus replication.

Conversely, we mapped the region within CLTC responsible for nsp3 binding using a series of deletion mutants (Figure 6D). Immunoprecipitation analysis revealed that the N‐terminal domain (1‐494 aa) of CLTC was necessary for its interaction with nsp3. However, the Distal Leg domain (495–1073 aa) also exhibited detectable binding (Figure 6E). The Proximal Leg & Hub domain showed no interaction. These results indicate that the primary interaction interface resides within the N‐terminal domain, while the Distal Leg may contribute to or stabilize the binding.

### 2.7 CLTC Knockout Suppresses Viral Proliferation by Inhibiting the Nucleation Stage of Autophagy

**FIGURE 7:**
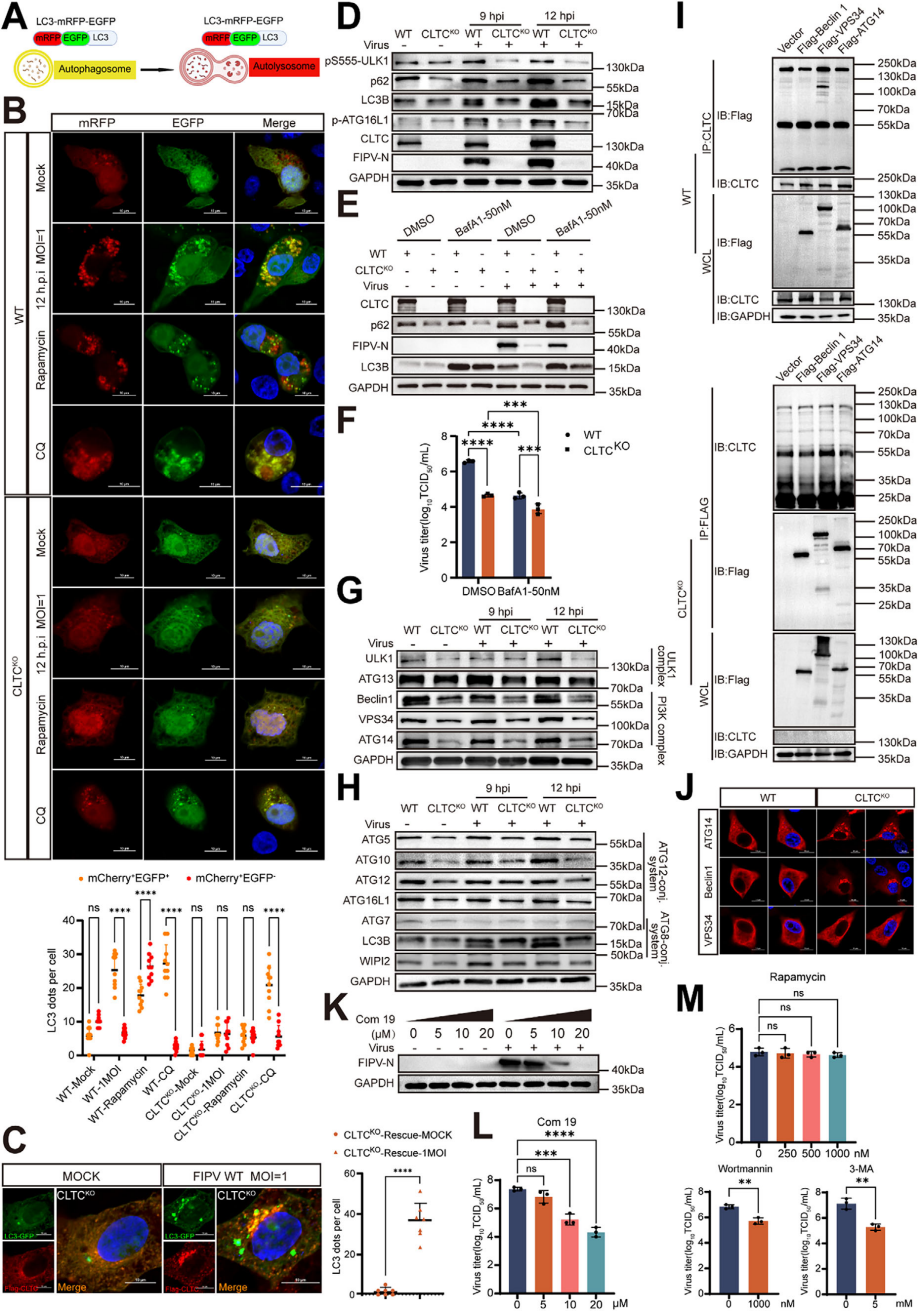
CLTC knockout suppresses viral proliferation by inhibiting the nucleation stage of autophagy. (A) Schematic diagram of LC3‐mRFP‐EGFP structure and the principle for probing autophagic flux using mRFP‐GFP‐LC3 construct. Under lysosomal acidification, GFP fluorescence is quenched, while mRFP remains stable. In autophagosomes (prior to lysosomal fusion), both GFP and mRFP fluoresce, producing yellow puncta (GFP^+^/mRFP^+^). In autolysosomes (post‐fusion), GFP signal dissipates, leaving red mRFP+ puncta (GFP^−^/mRFP^+^). Created with BioRender.com. https://BioRender.com/38gc36d (B) WT and CLTC KO cells were transfected with 2 µg of LC3‐mRFP‐EGFP for 24 h and then infected with FIPV WT (MOI = 1, 12 hpi) or treated with rapamycin (1000 nm) or CQ (50 µm). Scale bar = 10 µm. Representative confocal images are shown (Top). Quantification of LC3 puncta (yellow vs. red dots) was performed on randomly selected cells per condition (Bottom) (n = 10). Data are mean ± SD (unpaired two‐tailed Student's *t*‐test). ns: *p* ≥ 0.05; ^****^ *p* < 0.0001. (C) CRFK CLTC KO cells were transfected with 1 µg of LC3‐EGFP and 1 µg of pCAGGS‐Flag‐CLTC plasmid for 36 h and then infected with FIPV WT (MOI = 1, 12 hpi). Scale bar = 10 µm. Quantification of LC3 puncta was performed on randomly selected cells per condition (Right) (n = 7). Data are mean ± SD (unpaired two‐tailed Student's *t*‐test). ns: *p* ≥ 0.05; ^****^ *p* < 0.0001. (D, G, H) CRFK WT and CLTC KO cells were mock inoculated or infected with FIPV nsp3‐ZsGreen (MOI = 1) for 9, 12 h. (E, F) CRFK WT and CLTC KO cells were mock inoculated or infected with FIPV nsp3‐ZsGreen, and then treated with BafA1 (50 nm) (MOI = 1) for 12 h. Viral titers in the supernatants were quantified using a TCID_50_ assay on CRFK cells (n = 3). (I) Interaction between CLTC and the Class III PI3K complex. HEK293T cells were transfected with Flag‐tagged Beclin 1, VPS34, or ATG14 for 36 h. (Top) In WT cells, endogenous CLTC was immunoprecipitated (IP: CLTC), and the associated Flag‐tagged proteins were detected by immunoblotting (IB: Flag). (Bottom) As a specificity control, the same transfection and reverse immunoprecipitation (IP: Flag) were performed in CLTC KO cells. (J) WT and CLTC KO cells were transfected with Flag‐tagged ATG14, Beclin1, or VPS34 (red), and nuclei were stained with DAPI (blue). Scale bar = 10 µm. (K, L) CRFK cells were infected with FIPV nsp3‐ZsGreen (MOI = 1) or left uninfected, and treated with Com19 (0, 5, 10, 20 µm) for 12 h. (M) CLTC KO cells were infected with FIPV nsp3‐ZsGreen (MOI = 1) or left uninfected and treated with rapamycin (0, 250, 500, 1000 nm) for 12 h. CRFK WT cells were infected with FIPV nsp3‐ZsGreen (MOI = 1) or left uninfected, and treated with wortmannin (1000 µm), 3‐MA (5 mm) for 12 h. Western blot analysis was performed for the indicated proteins, with GAPDH as a loading control (D, E, G, H, I, K). Viral titers in the supernatants were quantified using a TCID_50_ assay on CRFK cells (n = 3) (F, L, M). Data are mean ± SD (one‐way ANOVA with Dunnett's test against the respective control group). ns: *p* ≥ 0.05; ^**^ *p* < 0.01; ^***^ *p* < 0.001; ^****^ *p* < 0.0001.

To investigate whether CLTC influences viral replication by modulating autophagy, we first employed a tandem fluorescent mRFP‐EGFP‐LC3 reporter system to monitor autophagic flux (Figure 7A). This construct allowed for the distinction between autophagosomes (yellow puncta, GFP^+^/mRFP^+^) and autolysosomes (red puncta, GFP^−^/RFP^+^) because of the different sensitivities of GFP and mRFP to lysosomal acidity [42].

In WT cells infected with FIPV WT, we observed a pronounced accumulation of yellow puncta, indicative of active autophagosome formation coupled with blocked lysosomal fusion—a phenotype mirroring that induced by chloroquine (CQ) treatment (Figure 7B and Figure S6A, B, F). Strikingly, this virus‐induced accumulation of yellow puncta was markedly suppressed in CLTC KO cells, while the relative proportion of red puncta increased, suggesting that CLTC deficiency impairs autophagosome biogenesis rather than the downstream flux (Figure 7B). Reconstitution of CLTC expression in KO cells restored the formation of LC3 puncta upon infection (Figure 7C).

We further examined the expression of key autophagy‐related proteins by Western blot. In WT cells infected with FIPV nsp3‐ZsGreen, the conversion of LC3‐I to lipidated LC3‐II increased over time (9 and 12 hpi), accompanied by the accumulation of p62, which is consistent with enhanced autophagosome formation but impaired lysosomal degradation (Figure 7D). In contrast, CLTC knockout substantially attenuated the infection‐driven accumulation of LC3‐II, further supporting the notion that CLTC deficiency suppresses autophagosome biogenesis (Figure 7D).

To distinguish between impaired autophagosome synthesis and enhanced degradation, we performed an autophagic flux assay using the lysosomal inhibitor Bafilomycin A1 (BafA1). First, cell viability assays confirmed that 50 nm BafA1 was non‐toxic to CRFK cells (Figure S6G). Western blot analysis revealed that while BafA1 caused robust LC3‐II accumulation in uninfected cells, it failed to induce further accumulation of LC3‐II in FIPV‐infected WT cells (Figure 7E), confirming that viral infection effectively blocks autophagic flux. Importantly, in infected CLTC KO cells, LC3‐II levels remained substantially reduced compared to WT controls even in the presence of BafA1. This inability to accumulate LC3‐II, even when lysosomal degradation is inhibited, definitively demonstrates that CLTC deficiency impairs the upstream nucleation of autophagosomes (Figure 7E). And the results of the TCID_50_ experiment also showed that the use of BafA1 could significantly inhibit viral proliferation (Figure 7F). Notably, CLTC knockout cells compared with WT cell, exhibited reduced autophagic flux upon FIPV nsp3‐ZsGreen infection compared with WT cells. To determine the stage at which autophagy is affected by CLTC, we assessed the expression of key regulators involved in the subsequent steps. Interestingly, CLTC knockout significantly downregulated core components of the class III PI3K complex—including ATG14, BECN1, and VPS34—which are essential for autophagosome initiation, suggesting that CLTC is required for the nucleation stage of autophagy (Figure 7G,H). Co‐immunoprecipitation confirmed a physical interaction between CLTC and these PI3K complex proteins (Figure 7I and Figure S7A, B). Furthermore, immunofluorescence revealed that CLTC deficiency caused aberrant aggregation of ATG14 and BECN1, indicating that CLTC is required to maintain the proper solubility and functional organization of the initiation machinery (Figure 7J and Figure S7C).

To functionally link impaired autophagy to reduced viral proliferation, we treated infected cells with various autophagy modulators. In WT cells, treatment with Com19, an inhibitor of the Beclin1‐ATG14L interaction, reduced viral titers in a dose‐dependent manner (0–20 µm) (Figure 7K,L; Figure S6C). Conversely, in CLTC KO cells, treatment with the autophagy initiation activator rapamycin at different concentrations failed to restore viral proliferation (Figure 7M and Figure S6A). Similarly, inhibiting autophagosome formation using wortmannin (1000 µm) or 3‐MA (5 mm) significantly decreased the production of infectious viral particles (Figure 7M and Figure S6D, E) in WT cells, phenocopying the effect observed in CLTC KO cells. Collectively, these data demonstrate that CLTC is essential for the nucleation stage of autophagy. CLTC deficiency disrupts the initial formation of autophagosomes, thereby restricting efficient FIPV proliferation.

## 3 Discussion

Positive‐sense single‐stranded RNA viruses remodel host membranes to form DMVs, which serve as platforms for viral replication [43]. However, a comprehensive understanding of this process has been impeded by two major technical limitations: the static nature of conventional imaging techniques, such as TEM, which cannot capture the dynamic process of DMV formation, and the lack of systems for functionally screening host factors recruited to DMVs under authentic infection conditions.

To overcome these challenges, we developed a live‐cell imaging system based on a recombinant FIPV expressing ZsGreen inserted into a hypervariable region of nsp3. For the first time, this approach enabled real‐time visualization of DMV dynamics and simultaneous tracking of nsp3 localization during early infection. The specificity of the nsp3‐ZsGreen signal for bona fide DMVs is strongly supported by IEM, which directly localized ZsGreen to these structures. Together with its spatiotemporal colocalization with established markers of replication organelles, such as dsRNA and ER‐resident proteins, and concordance with prior studies [13, 44, 45], these data validate our reporter as a faithful tool for studying DMV biogenesis. Beyond overcoming the limitations of static TEM, this system provides a more efficient and practical alternative to conventional immunofluorescence or TEM for DMV detection. Moreover, it enables the targeted identification of host factors associated with DMVs under physiologically relevant infection conditions, circumventing the need for in vitro‐induced DMV models or untargeted genome‐wide screens.

Our AP‐MS analysis utilizing this platform pinpointed CLTC as a host factor specifically associated with nsp3‐anchored DMVs. Functional assays established that CLTC is essential for the replication of various coronaviruses—such as human coronavirus 229E and representative viruses from β‐ and δ‐genera—highlighting a conserved dependency. This requirement appears selective, as CLTC was dispensable for VSV, PRV, or HSV replication, aligning with Liang et al.’s work and defining a coronavirus‐specific dependency [46]. These results underscore the specific and crucial role of CLTC in the coronavirus life cycle and affirm the utility of our system in identifying genuine DMV‐associated host factors.

Notably, the role of CLTC in coronavirus replication is distinct from its canonical function in endocytosis. CLTC knockout did not impair viral attachment or entry, likely due to compensatory upregulation of alternative entry pathways—such as very‐low‐density lipoprotein receptor (VLDLR), dynamin 3 (DNM3), and kinesin family member 5C (KIF5C)—as evidenced by our transcriptomic data. While these changes suggest a possible compensatory mechanism that allows the virus to bypass CLTC‐dependent entry, further functional validation is required to confirm this hypothesis [47, 48, 49]. Importantly, our data reveal a previously unappreciated role for CLTC in viral replication organelle formation. The specific interaction between nsp3 and CLTC, coupled with the recruitment of CLTC to DMV assembly sites, suggests a targeted mechanism whereby coronaviruses hijack host membrane remodeling machinery. In these biological processes, clathrin and other coat proteins like COPI/II are recruited by Arf1 to membranes, including the ER and Golgi, where they facilitate cargo sorting, trafficking, and membrane remodeling—processes often mediated by amphipathic helices [50, 51, 52, 53, 54]. It is well established that certain viral factors, such as the 3A proteins of poliovirus and coxsackievirus B3 [55, 56], can activate or recruit such cellular machinery to support viral replication, a strategy that parallels our finding of the specific nsp3–CLTC interaction.

**FIGURE 8:**
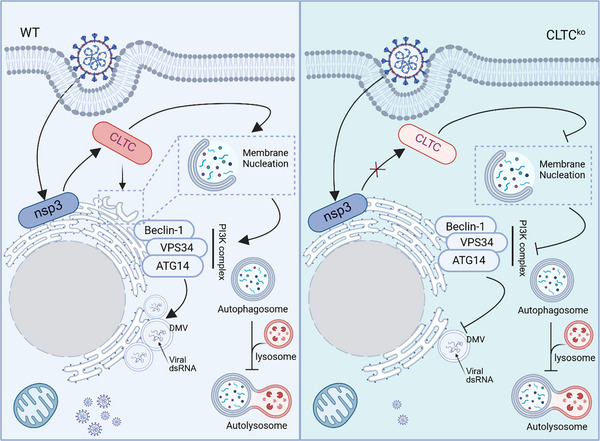
A model of CLTC‐mediated DMV biogenesis during coronavirus infection. In wild‐type cells, FIPV infection recruits CLTC to ER‐localized nsp3, facilitating DMV biogenesis and blocking autophagic flux to promote viral replication. In CLTC‐knockout cells, the loss of CLTC impairs autophagosome nucleation by reducing Beclin1–ATG14 complex expression. This disrupts the formation of autophagic precursor membranes, thereby preventing their hijacking by nsp3 for DMV assembly. Furthermore, partial restoration of autophagic flux severely restricts DMV biogenesis and viral replication. Created with BioRender.com. https://BioRender.com/6dygiyn.

Building on evidence linking CLTC to early autophagosome formation and the involvement of autophagy‐related proteins in DMV biogenesis [31, 57, 58], we propose a model (Figure 8) wherein nsp3 recruits CLTC to ER or ER‐derived membranes to promote curvature and generate autophagic precursor membranes. These CLTC‐structured platforms are subsequently hijacked by the virus to facilitate DMV assembly.

Although coronavirus‐induced vesicles are ER‐derived and share similarities with autophagosomes, the role of autophagy in DMV biogenesis has been controversial, with conflicting reports regarding ATG5‐dependence [58, 59, 60]. Previous proposals that DMVs may result from viral hijacking of autophagy pathways, along with observations that certain viruses, such as porcine reproductive and respiratory syndrome virus (PRRSV), exploit accumulated autophagosomes as replication sites, highlight the complex interplay between autophagy and viral replication [61, 62]. Our study provides crucial mechanistic insight into this relationship by demonstrating that CLTC supports DMV formation through specific regulation of autophagy nucleation. We found CLTC essential for this autophagosome initiation, as its loss significantly downregulates BECN1, ATG14, and VPS34, key components of the class III PI3K complex and impairs autophagosome nucleation. This finding aligns with previous observations in SARS‐CoV‐2 nsp3/4 expression systems, confirming that this mechanism is conserved and critical during live viral infection [57, 63]. As Beclin1 and ATG14 drive phosphatidylinositol‐3‐phosphate (PI3P) production and recruit membranes to autophagic precursors [64, 65], our findings indicate that CLTC deficiency disrupts DMV biogenesis by impairing the formation of the very autophagic precursor membranes that coronaviruses hijack for their replication organelles.

We also observed that FIPV infection arrests autophagic flux, consistent with reports that coronaviruses block autophagosome–lysosome fusion [66, 67]. This blockade likely represents an active strategy to freeze autophagic membranes at the expansion stage for DMV assembly, protecting them from lysosomal degradation. Furthermore, autophagy initiation activators failed to restore autophagic flux or viral proliferation in CLTC KO cells, indicating that CLTC acts upstream in the formation of these hijacked precursor membranes rather than simply consuming downstream autophagosomes. The parallel involvement of both CLTC and VMP1 in modulating the autophagy–apoptosis balance in other biological contexts suggests these factors may represent nodes in a broader network of membrane remodeling regulation that coronaviruses exploit [68, 69].

Our study has several limitations that present opportunities for future research. First, the precise structural basis of the nsp3–CLTC interaction remains unresolved. Although our mapping and modeling defined the critical domains, high‐resolution structural delineation (e.g., by cryo‐EM) is required to elucidate the exact intermolecular contacts and understand how nsp3 recruits and manipulates the host membrane curvature machinery. Second, while our validation in Huh‐7 cells with HCoV‐229E confirms CLTC's role in a human hepatic model, extending these findings to other physiologically relevant human cell types (e.g., respiratory epithelial cells) infected with a broader panel of human‐tropic coronaviruses would further strengthen the clinical and translational relevance of our findings.

In conclusion, we have developed a live‐cell imaging platform that enables real‐time tracking of nsp3 and direct observation of DMV dynamics during early coronavirus infection. This system provides a robust and versatile tool for systematically identifying host factors involved in DMV biogenesis and for screening early‐stage antiviral compounds. Beyond this methodological advance, we uncover an unexpected role for CLTC in coronavirus replication. We demonstrate that CLTC is selectively hijacked by the virus to promote autophagosome nucleation, a function not previously recognized in viral infection. Mechanistically, CLTC mediates membrane curvature and initiates the formation of autophagic precursors, which are subsequently co‐opted by nsp3 to scaffold DMV assembly and facilitate viral RNA synthesis. Our findings expand the repertoire of host factors involved in coronavirus replication organelle formation—including VMP1 and TMEM41B—and establish CLTC as a crucial regulator of autophagosome nucleation during infection. These insights not only advance our mechanistic understanding of DMV biogenesis but also nominate CLTC and the autophagosome nucleation pathway as potential therapeutic targets for antiviral intervention.

## 4 Methods

### 4.1 Cell Culture and Virus

CRFK cells were kindly provided by Anding Zhang at Huazhong Agricultural University, Wuhan, China. HEK293T cells were purchased from the Cell Bank of the Chinese Academy of Sciences (Shanghai, China). CRFK and HEK293T cells were cultured and maintained in Dulbecco's modified Eagle's medium/high glucose (DMEM; Gibco, #11965092). All media were supplemented with 10% heat‐inactivated fetal bovine serum (FBS; Gibco, #10091148) and 1% penicillin‒streptomycin (Sigma, #P4333), and the cells were cultured in a humidified incubator at 37°C with 5% CO_2_. The viruses FIPV 79–1146 strain (GenBank: MW030109.1), FIPV QS‐79 strain (GenBank: MW030108.1), PDCoV strain CHN‐HN‐2014 (GenBank: KT336560), PEDV strain YN200 (GenBank: KT021233), PEDV strain CTP10 (GenBank 282 JQ023162.1), and MHV A59 strain (GenBank: MF618253.1) were stored in our laboratory. VSV and pseudorabies virus (PRV) were generously provided by Professor Gang Cao (Hubei, China). The PEDV strain CTP10 was generously provided by Professor Yongchang Cao (Sun Yat‐Sen University, China). FIPV was propagated in CRFK cells. TGEV, VSV, and PRV were propagated in PK‐15 cells. PEDV and PDCoV were propagated in Vero E6 cells and LLC‐PK1 cells, respectively. MHV was propagated in L929 cells. For the diverse coronavirus antiviral assay (Figure 4k), CRFK WT and CLTC KO cells were infected with the indicated viruses (FIPV, TGEV, PEDV, MHV, PDCoV) to evaluate viral replication in a uniform cellular background.

### 4.2 Plasmid Construction

The pKLV2‐U6gRNA5 (BbsI) ‐PGKpuro2AZsG‐W plasmid (Addgene, #67975) was generously provided by Professor Shengsong Xie (Huazhong Agricultural University, China). The ZsGreen gene was from the pKLV2‐U6gRNA5 (BbsI) ‐PGKpuro2AZsG‐W (Addgene, #67975) plasmid, and the FIPV nsp3 173–180aa was replaced with ZsGreen tag in the FIPV‐BAC plasmid. Full‐length cDNA encoding CLTC, BECN1, ATG14, and VPS34 was amplified by RT‒PCR from total RNA extracted from CRFK cells. The CLTC plasmid was constructed in pCAGGS with a Flag‐tag or an HA‐tag in the N‐terminal region. The BECN1, ATG14, and VPS34 plasmids were constructed in pCAGGS with a Flag‐tag in the N‐terminal region. The pCAGGS‐HA‐FIPV nsp3, pCAGGS‐Flag‐FIPV nsp4, and pCAGGS‐Strep EGFP‐FIPV nsp3‐4‐His plasmids were used to express codon‐optimized nsp3, nsp4, and nsp3–4, respectively, and contain HA‐tag, Flag‐tag, EGFP‐tag, and Strep‐tag at the N‐terminus or His‐tag at the C‐terminus. The cDNA fragments encoding CLTC and nsp3 truncation mutants were cloned into the pcDNA3.1‐His‐tag vector and the pCAGGS‐HA‐tag vector, respectively. For rescue experiments, to abolish cleavage by sgRNA and Cas9 in CLTC‐KO cells, specific bases in the protospacer adjacent motif sequence of CLTC that did not alter the encoded amino acid were mutated. The mutant sequence was cloned into the pCAGGS‐Flag vector. Sanger sequencing (Tsingke) was performed to confirm the sequence of all plasmids. All primers used for plasmid construction are shown in Table 1.

**TABLE 1:** Primers used for cloning.

Primer Name	Sequence (5′ to 3′)
FI3‐sgRNA1‐Fwd	GGCGATGTAATTGTCATTGC
FI3‐sgRNA1‐Rev	AAAAGCACCGACTCGGTGCCACTTTTTCAAGTTGATAACGGACTAGCCTTATTTTAACTTGCTATTTCTAGCTCTAAAAC
FI3‐sgRNA2‐Fwd	GGTTTTAGTTAAAACGTTTG
FI3‐sgRNA2‐Rev	AAAAGCACCGACTCGGTGCCACTTTTTCAAGTTGATAACGGACTAGCCTTATTTTAACTTGCTATTTCTAGCTCTAAAAC
79‐ORF3‐sgRNA1‐Fwd	GGTAACATTACACAGGCTTT
79‐ORF3‐sgRNA1‐Rev	AAAAGCACCGACTCGGTGCCACTTTTTCAAGTTGATAACGGACTAGCCTTATTTTAACTTGCTATTTCTAGCTCTAAAAC
79‐ORF3‐sgRNA2‐Fwd	GGTGTAACTAAACTTTCAAA
79‐ORF3‐sgRNA2‐Rev	AAAAGCACCGACTCGGTGCCACTTTTTCAAGTTGATAACGGACTAGCCTTATTTTAACTTGCTATTTCTAGCTCTAAAAC
FI3‐de173‐180‐ty1‐Fwd	GGCGATGTAATTGTCATTGCTGGATACACCTTTTACAAGG
FI3‐de173‐180‐ty1‐Rev	ATTTTCTTCTACGAGCGAAACTTGTTCAACAAAGTCTTCAACATCG
FI3‐de173‐180‐ty2‐Fwd	GTTTCGCTCGTAGAAGAAAATATGGCCCAGTCCAAGCACGGCCTGACCAA
FI3‐de173‐180‐ty2‐Rev	GAGGAAAGTTGCTCATCAGGGGGCAAGGCGGAGCCGGAGGCGAT
FI3‐de173‐180‐ty3‐Fwd	GCAACTTTCCTCAGTAGAAAAAAAAGATGAAGTCTCTGC
FI3‐de173‐180‐ty3‐Rev	ACACCCTCCTCCACAAACGTTTTAACTAAAACCTTTTGTGCTG
79‐ORF3 ZSgreen‐Fwd	ACTAAATTTAAAGTTAAGGATGGCCCAGTCCAAGCACGG
79‐ORF3 ZSgreen‐Rev	GAGAAGAGGCTGCATTGTAATTAGGGCAAGGCGGAGCCGG
pCAGS‐HA‐CLTC‐Fwd	GAATTCGAGCTCATCGATGGTACCATGGCCCAGATTCTGCCA
pCAGS‐HA‐CLTC‐Rev	AAGATCCTTAATTAATTAAGATCTCATGCTGTACCCAAAGCCAG
FLAG‐CLTC‐RES‐1‐Fwd	AAGGATGACGACGATAAGGAATTCATGGCCCAGATTCTGCCA
FLAG‐CLTC‐RES‐1‐Rev	TACTCGCCTAGATGTACAAGTGTAGATGCCAAGCGTCCAAA
FLAG‐CLTC‐RES‐2‐Fwd	ACTTGTACATCTAGGCGAGTATCAGGCAGCTGTTGATGG
FLAG‐CLTC‐RES‐2‐Rev	CTCGAGGCATGCCCGGGTACCCTACATGCTGTACCCAAAGCCAG
FLAG‐BECN1‐Fwd	GACGATAAGGAATTCGAGCTCATGGAGGGGTCTAAAACGTCC
FLAG‐BECN1‐Rev	CTCGAGGCATGCCCGGGTACCTCATTTGTTATAAAACTGTGAGGAT
FLAG‐ATG14‐Fwd	GACGATAAGGAATTCGAGCTCATGGCGTCTCCCAGTGGGAA
FLAG‐ATG14‐Rev	CTCGAGGCATGCCCGGGTACCTTAGCGGTGTCCAGTGTAAGC
FLAG‐VPS34‐Fwd	GACGATAAGGAATTCGAGCTCATGGGAGAAGCGGAGAAGT
FLAG‐VPS34‐Rev	CTCGAGGCATGCCCGGGTACCTCATTTTCTCCAGTACTGGGC

### 4.3 Antibodies and Reagents

Primary antibodies used for western blot and immunofluorescence staining included rabbit polyclonal anti‐CLTC (Abcam: AB21679; 1:1000), mouse anti‐dsRNA J2 (English & Scientific Consulting, 10010200; 1:1000), mouse monoclonal anti‐GAPDH (Proteintech: 60004‐1‐Ig; 1:5000), mouse monoclonal anti‐β‐actin (Proteintech: 66009‐1‐Ig; 1:5000), mouse monoclonal anti‐HA (MBL: M180‐3; 1:10,000), rabbit monoclonal anti‐HA (Cell Signaling Technology: 3724S; 1:1000), mouse monoclonal anti‐Flag (Proteintech: 66008‐4‐Ig;1:5000), rabbit polyclonal anti‐Flag (Proteintech: 20543‐1‐AP; 1:1000), rabbit monoclonal anti‐Phospho‐ULK1 (Ser555) (Cell Signaling Technology: 5869T; 1:1000), rabbit polyclonal anti‐SQSTM1/p62 (Abcam: AB314504; 1:1000), rabbit monoclonal anti‐LC3B (Abcam: AB192890; 1:1000), mouse monoclonal anti‐ZsGreen (Origene:TA180002; 1:2000), rabbit monoclonal anti‐ULK1 (Cell Signaling Technology: 8054T; 1:1000), rabbit monoclonal anti‐ATG13(Abcam: AB201467; 1:1000), rabbit monoclonal anti‐Beclin1(Abcam: AB207612; 1:2000), rabbit monoclonal anti‐ATG14(Abcam: AB315009; 1:1000), rabbit monoclonal anti‐VPS34 (Cell Signaling Technology: 4263S; 1:1000), rabbit monoclonal anti‐ATG5 (Cell Signaling Technology: 12994S; 1:1000), rabbit monoclonal anti‐ATG12 (Cell Signaling Technology: 4180T; 1:1000), rabbit monoclonal anti‐ATG16L1 (Cell Signaling Technology: 8089S; 1:1000), rabbit monoclonal anti‐ATG7 (Cell Signaling Technology: 8558T; 1:1000), rabbit monoclonal anti‐ATG16L1 (phospho S278) (Abcam: AB195242; 1:1000), rabbit monoclonal anti‐ATG10 (ABclonal: A6848; 1:500), rabbit polyclonal anti‐WIPI2 (ABclonal: A7537; 1:500). The secondary antibodies used included Alexa Fluor 594 Goat Anti‐Mouse IgG (H + L) (Invitrogen: A‐11005; 1:1000), Alexa Fluor 594 Anti‐Rabbit IgG (H + L) (Invitrogen: A‐11012; 1:1000), Alexa Fluor 488 Anti‐Mouse IgG (H + L) (Invitrogen: A‐11001; 1:1000) and Alexa Fluor 488 Anti‐Rabbit IgG (H + L) (Invitrogen: A‐11008; 1:1000). The following reagents were used in this study: puromycin (Abcam: Ab141453), polybrene (R&D Systems: 7711), rapamycin (MCE: HY‐10219), chloroquine (CQ) (MCE: HY‐17589A), Beclin1‐ATG14L interaction inhibitor 1 (com 19) (MCE: HY‐156237), 3‐methyladenine (3‐MA) (MCE: HY‐19312), wortmannin (MCE: HY‐10197).

### 4.4 Generation of CLTC‐Knockout and Other Gene Knockout Pool CRFK Cell Lines

Single guide RNA (sgRNA) sequences targeting CLTC and another 20 genes were cloned into the lentiCRISPR v2 vector. Lentiviruses were generated by cotransfecting HEK293T cells with the above plasmids, the packaging plasmid psPAX2, and the membrane plasmid pMD2.G using Lipofectamine 2000. Lentiviral supernatants were collected at 72 h post‐transfection, filtered (0.45 µm), and stored at −80°C. CRFK cells were infected with lentiviruses carrying CLTC sgRNA in the presence of 8 µg/ml polybrene, and positive clones were selected with puromycin (5.5 µg/ml) for 48–60 hpi. Monoclonal cells were isolated using the limiting dilution method in 96‐well plates, expanded, and verified via western blotting and sequencing. All sgRNAs used for constructing knockout cells are shown in Table 2.

**TABLE 2:** sgRNAs used for constructing knockout cells.

Primer Name	Sequence (5′ to 3′)
CLTC‐Fwd	CACCGGATATTCACCCAGGTGAACG
CLTC‐Rev	AAACCGTTCACCTGGGTGAATATCC
FLNB‐Fwd	CACCGTGGGACAGGTACATCATCGT
FLNB‐Rev	AAACACGATGATGTACCTGTCCCAC
FN1‐Fwd	CACCGTAACCAGTAATCCTGGCACG
FN1‐Rev	AAACCGTGCCAGGATTACTGGTTAC
DEK‐Fwd	CACCGACATGCTGTCGACCTGCGGG
DEK‐Rev	AAACCCCGCAGGTCGACAGCATGTC
RUVBL1‐Fwd	CACCGACAGACGTGATACTCACCAA
RUVBL1‐Rev	AAACTTGGTGAGTATCACGTCTGTC
CCT7‐Fwd	CACCGTTCAACAGGATAACTGGTGT
CCT7‐Rev	AAACACACCAGTTATCCTGTTGAAC
RACK1‐Fwd	CACCGGCCATAATTGGTCTCATCCC
RACK1‐Rev	AAACGGGATGAGACCAATTATGGCC
RPS2‐Fwd	CACCGTACCCTGCAAGGTAGGCCGG
RPS2‐Rev	AAACCCGGCCTACCTTGCAGGGTAC
SLC30A7‐Fwd	CACCGTTGTCCGACAGGATAGACCT
SLC30A7‐Rev	AAACAGGTCTATCCTGTCGGACAAC
CDKL5‐Fwd	CACCGTCTAGAAAATGAAGAAGTCA
CDKL5‐Rev	AAACTGACTTCTTCATTTTCTAGAC
CORO6‐Fwd	CACCGGAACCCATCATTACACTCGA
CORO6‐Rev	AAACTCGAGTGTAATGATGGGTTCC
RIC8A‐Fwd	CACCGGAGGTCGGGACCGGCGTGCA
RIC8A‐Rev	AAACTGCACGCCGGTCCCGACCTCC
MYO5A‐Fwd	CACCGAGTTTGGATACCTGATCCTG
MYO5A‐Rev	AAACCAGGATCAGGTATCCAAACTC
XRCC6‐Fwd	CACCGGGGCACCTTCCGTTTATCGT
XRCC6‐Rev	AAACACGATAAACGGAAGGTGCCCC
CHD3‐Fwd	CACCGGCCGTACGACCAGTCCACGT
CHD3‐Rev	AAACACGTGGACTGGTCGTACGGCC
PCM1‐Fwd	CACCGTATCACGTCTGAACTAAACG
PCM1‐Rev	AAACCGTTTAGTTCAGACGTGATAC
CEBPB‐Fwd	CACCGCGCGTTCATGCAACGCCTGG
CEBPB‐Rev	AAACCCAGGCGTTGCATGAACGCGC
MYO1E‐Fwd	CACCGTTATGTTCACAGGGAAGCAA
MYO1E Rev	AAACTTGCTTCCCTGTGAACATAAC
MAP1B‐Fwd	CACCGGCAGATTCGGATCCAATTAG
MAP1B‐Rev	AAACCTAATTGGATCCGAATCTGCC
MACF1‐Fwd	CACCGTTCATCGAGAAATCCCGCAG
MACF1‐Rev	AAACCTGCGGGATTTCTCGATGAAC
CALM3‐Fwd	CACCGCTGACCGTCCCCGTCGATGT
CALM3‐Rev	AAACACATCGACGGGGACGGTCAGC

### 4.5 Cell Viability Assay

Cell viability was assessed according to the instructions provided for the CellTiter 96 Aqueous One Solution Cell Proliferation Assay (MTS) (Promega, G3580). Cells were seeded into 96‐well plates and incubated for 24 h. Twenty microliters of CellTiter 96 AQueous One Solution Reagent was added to each well of the 96‐well assay plate containing the samples in 100 µL of culture medium and incubated at 37°C for 4 h in standard culture conditions. The plate was briefly shaken, and the absorbance of the treated and untreated cells was measured using a plate reader at an optical density (OD) of 490 nm.

### 4.6 High‐Throughput Microscopy and Image Analysis

CRFK cells were seeded in 96‐well plates and then infected with FIPV nsp3‐ZsGreen (MOI = 10). After infection for 4 hpi, the cells were loaded into a high‐content imaging system (Opera Phenix, PE, USA) to track the dynamic formation of DMVs. Images were acquired every 15 min for a duration of 18 h (37°C, 5%CO2).

### 4.7 Affinity Purification Mass Spectrometry (AP‒MS)

CRFK cells that were mock inoculated or infected with FIPV WT, FIPV deORF3‐ZsGreen, or FIPV nsp3‐ZsGreen (MOI = 5, 9 hpi) were lysed using IP lysis buffer supplemented with protease inhibitors. The clarified lysates were then subjected to ZsGreen‐Trap immunoprecipitation and subsequent mass spectrometric analysis.

### 4.8 RNA Isolation and RT‒qPCR

Cells were homogenized in TRIzol reagent (Thermo Fisher Scientific, #15596018), and RNA was extracted through phase separation with chloroform (Thermo Fisher Scientific, #C/4960/PB08), after which the RNA was precipitated with isopropanol (Sigma, #I9516) and washed with ethanol. The RNA pellet was then resuspended in DEPC water (Thermo Fisher Scientific, #R0601). cDNA was subsequently synthesized from the purified RNA using a SuperScript IV reverse transcription kit (Thermo Fisher Scientific, #18091050) or a HiScript II 1st Strand cDNA Synthesis Kit (Vazyme, # R211‐01). The qPCR mixture was prepared with FastStart Universal SYBR Green Master Mix (ABclonal, RK21203), specific primers (Table 3), and a cDNA template, and amplification was performed under the optimized cycling conditions on a CFX384 Touch Real‐Time PCR System. Data were analyzed using Bio‐Rad CFX Manager software.

**TABLE 3:** Primers used for viral load quantification.

Species/Virus	Primer Name	Sequence (5’‐3’)
FIPV nsp3‐ZsGreen	S‐Fwd	AGCACAGGTTGTTGTGGATGC
	S‐Rev	ACAGCAAAGTATGCACGGC
	E‐Fwd	AGTACTACCTGCACGCCATG
	E‐Rev	GGCACAGTAGCGTTCACCATA
	M‐Fwd	AACTACTGCCACAGGATGGG
	M‐Rev	TTCATCTCCCCAGTTGACGC
	N‐Fwd	CCTGCCAAAGGATGATGCCA
	N‐Rev	GCCTCCACAAGAGTTACAGACA
	‐vRNA N‐Fwd	GCAAGGGAGATGTGACAACTTTC
	‐vRNA N‐Rev	AGCTATCTGAGGGTAGCATTTGG
	+vRNA N‐Fwd	GATGAACCTTCCAAAAGACGTGG
	+vRNA N‐Rev	AGATCCTTGTTCGAGGGTAATGG
Felis catus	RPS7‐Fwd	GTCCCAGAAGCCGCACTTTGAC
	RPS7‐Rev	CTCTTGCCCACAATCTCGCTCG

### 4.9 Plaque Assay

Monolayers of cells were infected with FIPV nsp3‐ZsGreen (MOI = 0.1) for 1 h and then washed with PBS (Sigma, #D8662) before the addition of 1 mL of prewarmed overlay (2% methylcellulose (Sigma, #C5013): propagation media containing 2% FBS at a ratio of 2:3). At 24 hpi, the cells were fixed with 4% formaldehyde (Sigma, #F8775) and stained with crystal violet solution (HistoLab, CL.42555) after the overlay was removed. Plaques were manually counted.

### 4.10 Western Blot

Cells were lysed in cell lysis buffer for western blotting and IP (Beyotime, P0013) or mammalian cell lysis buffer (CWBIO, CW0889) supplemented with protease inhibitors (Roche, 04693132001). The lysates were clarified by centrifugation, and equal amounts of protein were separated by SDS‒PAGE and transferred onto nitrocellulose membranes (GE Healthcare Life Sciences, 10600001 and 10600002). The membranes were blocked with 5% bovine serum albumin (BSA; Sigma, A1933) in Tris‐buffered saline with Tween (TBST; Sigma, SRE0031) and incubated with primary antibodies, followed by incubation with HRP‐conjugated secondary antibodies. The protein bands were visualized using SuperPico ECL Master Mix (Vazyme, E432‐01) and an ECL detection system (Amersham Biosciences, NJ, USA). All the bands were detected within the linear range of the system.

### 4.11 Immunoprecipitation Assay

HEK293T cells were lysed with radioimmunoprecipitation assay (RIPA) lysis buffer IP lysis buffer [10 mm HEPES (pH 7.5), 150 mm KCl, 3 mm MgCl2, 0.5% NP‐40], 0.1 mm phenylmethylsulfonyl fluoride (PMSF), and protease inhibitor] for 30 min and then subjected to centrifugation at 12 000×g for 10 min at 4°C. The supernatants were immunoprecipitated with the appropriate antibodies at 4°C overnight. The immune complexes were incubated with protein A/G‐agarose beads for 2 h, washed five times with lysis buffer, and eluted in 1×SDS‒PAGE sample buffer for western blot analysis.

### 4.12 Viral Attachment and Internalization Assay

WT and CLTC KO cells were infected with FIPV nsp3‐ZsGreen (MOI = 10) and incubated at 4°C for 1 h. For the virus attachment assay, the cells were washed three times with cold PBS (at 4°C) to remove unbound viral particles, and the freeze‐thawed cell lysate was harvested to determine the viral titer. For the internalization assay, cells infected as described above were further cultured with prewarmed DMEM at 37°C for another hour. Subsequently, the cells were treated with 1 mg/mL pronase in cold PBS to remove the attached but uninternalized viral particles. After being washed three times, the frozen‐thawed cell lysate was harvested to determine the viral titer.

### 4.13 Viral Replication and Release Assay

CRFK cells were infected with FIPV nsp3‐ZsGreen in 12‐well plates. Afterward, the cells were washed with PBS 3 times. The cell frozen‒thawed lysate and supernatant were harvested to determine the viral titer in the cells (intracellular) and culture media (extracellular).

### 4.14 Immunofluorescence and Confocal Microscopy Analysis

CRFK or HEK293T cells were plated on coverslips in confocal dishes and infected with FIPV nsp3‐ZsGreen or transfected with the indicated plasmids. At the specified time points after infection or transfection, the cells were fixed with 4% paraformaldehyde for 15 min, permeabilized with 0.2% Triton X‐100 (Sigma–Aldrich, X100) in PBS for 10 min, and blocked with 4% BSA at room temperature for 1 h. Cells were then incubated with the corresponding primary antibodies at 4°C overnight, followed by incubation with Alexa Fluor FITC‐, 488‐, or 594‐conjugated goat anti‐mouse or anti‐rabbit secondary antibodies for 1 h at room temperature. Nuclei were stained with DAPI (Invitrogen, 00–4959‐52) for 5 min. After each step, the cells were washed five times with PBS. Confocal images were acquired using a Nikon AXR NSPARC confocal microscope, and the data were analyzed using NIS‐Elements software. ImageJ and Fiji were used for statistical analysis of the images.

### 4.15 Transmission Electron Microscopy

CLTC KO and CRFK cells were mock inoculated or infected with FIPV nsp3‐ZsGreen at a series of MOIs at different time points. The cells were subsequently washed three times with precooled PBS and fixed by adding 3 mL of 2.5% glutaraldehyde (ServiceBio) at room temperature for 2 h. After fixation, the cells were transferred to a 2 mL centrifuge tube. TEM was performed by ServiceBio Co., Ltd., and images were taken using the HZAU TEM platform (no. H7650, HITACHI).

### 4.16 Immunogold Staining and Electron Microscopy

CLTC‐KO and CRFK cells were mock inoculated or infected with FIPV nsp3‐ZsGreen (MOI = 5, 9 hpi). The cells were subsequently washed three times with precooled PBS and fixed with a mixture of 4% PFA and 0.05% glutaraldehyde prepared in 0.2 M HEPES buffer for 10 min (room temperature) and then with 4% PFA alone for 30 min (room temperature). After fixation, the cells were transferred to a 2 mL centrifuge tube. All the samples were then subjected to immunogold staining and transmission electron microscopy analysis by ServiceBio Co., Ltd.

### 4.17 Confocal Microscopy Observation of Autophagy

For autophagy evaluations, cells were seeded in confocal culture dishes and transfected with the autophagy dual‐labeled plasmid LC3‐mRFP‐EGFP for 24 h prior to treatment and then infected with FIPV nsp3‐ZsGreen (MOI = 1, 12 hpi). LC3 puncta were observed, and images were captured using a confocal microscope.

### 4.18 Molecular Docking and Structural Analysis

The initial 3D structures of FIPV nsp3 and host CLTC were retrieved from the AlphaFold Protein Structure Database (https://alphafold.com/). The raw PDB files were imported into the Molecular Operating Environment (MOE 2019.1) platform for pre‐processing. This included the removal of solvent molecules and ions, protonation using the Protonate 3D module, and completion of missing atoms or side chains. The structures were subsequently subjected to energy minimization to relieve steric clashes. Using HADDOCK (High Ambiguity Driven protein‐protein DOCKing) software, the protein was set to be rigid, and the docking contact site was set to be full surface. After docking, the conformation with the lowest binding energy was selected and visualized using PyMOL.

### 4.19 Statistical Analysis

All statistical analyses were performed using GraphPad Prism v8.0. Tests included unpaired two‐tailed Student's *t*‐test, one‐way ANOVA, or two‐way ANOVA, as specified in the respective figure legends. The results are shown as mean ± SD, with individual data points representing parallel replicates. Significance thresholds: ns: p ≥ 0.05; ^*^ *p* < 0.05; ^**^ *p* < 0.01; ^***^ *p* < 0.001; ^****^ *p* < 0.0001.

## Author Contributions

G.P. and J.X. did conceptualization. J.X., H.L., Z.J., D.Z., and Y.S. did methodology. P.L., Z.S., S.N., and J.Z. did resource. J.X. and H.L. did data analysis. J.X. did writing – original draft. J.X., H.L., and G. P. did writing – review and editing. G.P. did funding acquisition. G.P. did supervision.

## Funding

This work was supported by grant from National Funds for Distinguished Young Scientists of China (Grant 32125037, G.P.) and the Hubei Natural Science Foundation Innovation Group, China (Grant 2022CFA027, G.P.).

## Conflicts of Interest

The authors declare no conflicts of interest.

## Supporting information




**Supporting File**: advs74450‐sup‐0001‐SuppMat.docx.

## Data Availability

The data that support the findings of this study are available from the corresponding author upon reasonable request.
