# Supplementary material for: Coronavirus Nsp3 Hijacks CLTC to Modulate Autophagosome Nucleation for Promoting DMV Formation and Viral Replication
Source: Adv Sci (Weinh). 2026 Feb 21;13(24):e21626. doi: 10.1002/advs.202521626 (PMC13116158; doi:10.1002/advs.202521626)
Supplement: Supplementary file 1 — Supporting File: advs74450‐sup‐0001‐SuppMat.docx. [file ADVS-13-e21626-s001.docx]

Supporting Information

Coronavirus nsp3 hijacks CLTC to modulate autophagosome nucleation for promoting DMV formation and viral replication

Juan Xu, Hang Li, Peng Liu, Zhe Jiao, Ding Zhang, Zhelin Su, Sai Niu, Jintao Zhang, Yuejun Shi, and Guiqing Peng*


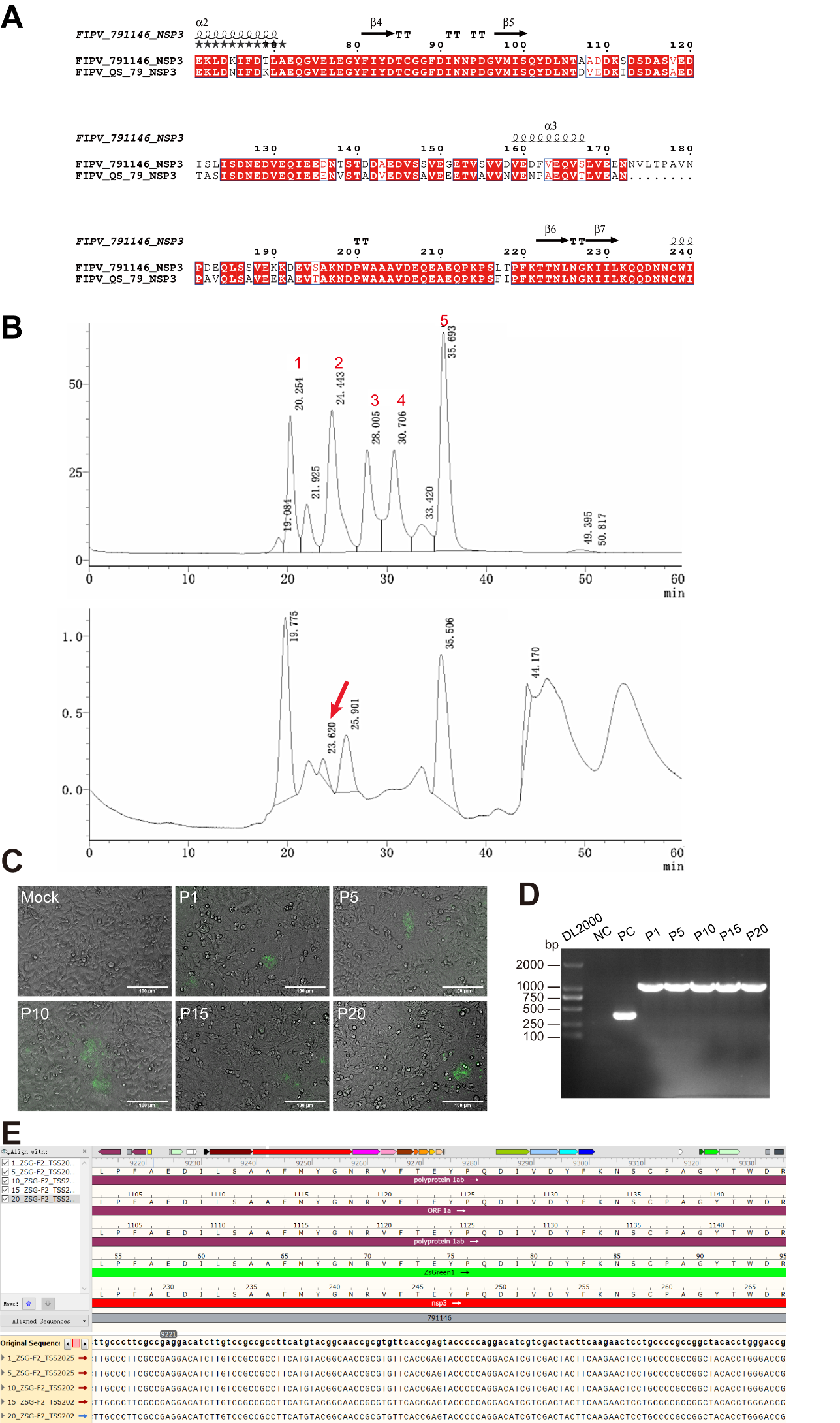


**Figure S1** | Construction and validation of the FIPV nsp3-ZsGreen virus. (A) Sequence alignment of FIPV nsp3 from FIPV QS-79 and FIPV 79-1146. Deletion of amino acid residues 173–180 was observed in Type I nsp3 (indicated by dashed lines). (B) FSEC analysis of protein fusion expression in the supernatant of CRFK cells infected with FIPV nsp3-ZsGreen (MOI = 1, 24 hpi). Upper panel: Representative chromatogram of molecular weight standards: 1: Thyroglobulin 670 kDa; 2: γ-globulin 158 kDa; 3: Ovalbumin 44 kDa; 4: Myoglobin 17 kDa; 5: Vitamin B12 1.35 kDa. (C) Fluorescence microscopy of CRFK cells infected with the nsp3-ZsGreen reporter virus at different passages (P1, P5, P10, P15, and P20), showing consistent ZsGreen signal. (D) PCR analysis of the nsp3-ZsGreen junction region across passages (P1–P20). A single band of the expected size (~1000 bp) was detected in all passages, with no smaller band indicative of deletion mutants. (E) Sanger sequencing confirmation of the nsp3-ZsGreen insertion site. Sequence alignment of PCR products from passages P1–P20 showed 100% identity to the reference genome at the ZsGreen insertion site and within the flanking nsp3 region.


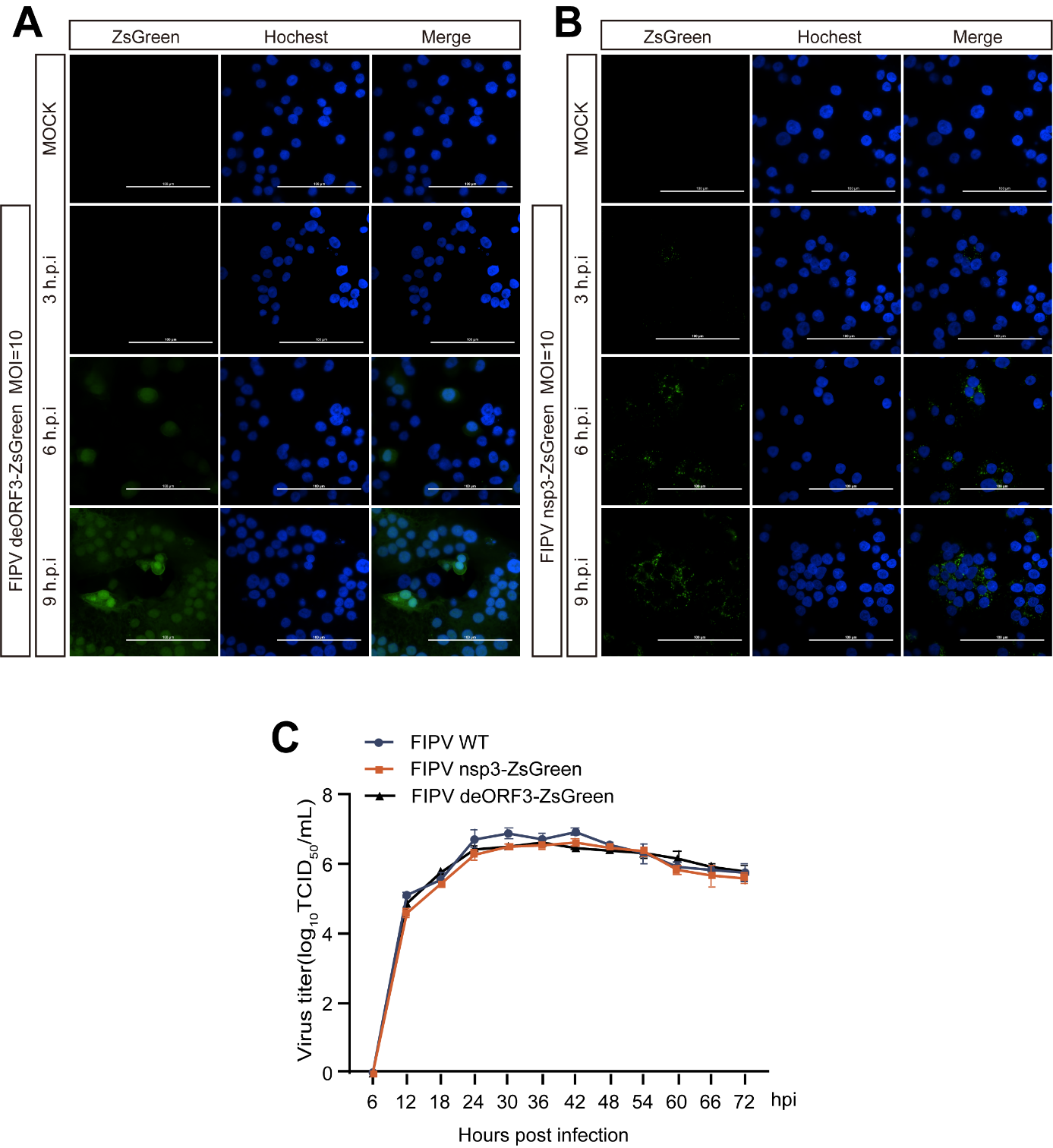


**Figure S2** | Live-cell imaging of CRFK cells infected with FIPV deORF3-ZsGreen and FIPV nsp3-ZsGreen. (A, B) Live-cell imaging of CRFK cells infected with FIPV deORF3-ZsGreen(A) or FIPV nsp3-ZsGreen(B) (MOI = 10) at 3, 6, 9 hpi. Scale bar = 10 μm. (C) Multistep growth curves of FIPV deORF3-ZsGreen, FIPV nsp3-ZsGreen or FIPV WT after infection (MOI = 0.1) of CRFK cells. The infectious virus titer sampled at the indicated time points was determined by end-point dilution in CRFK cells and calculated as TCID_50_/mL.


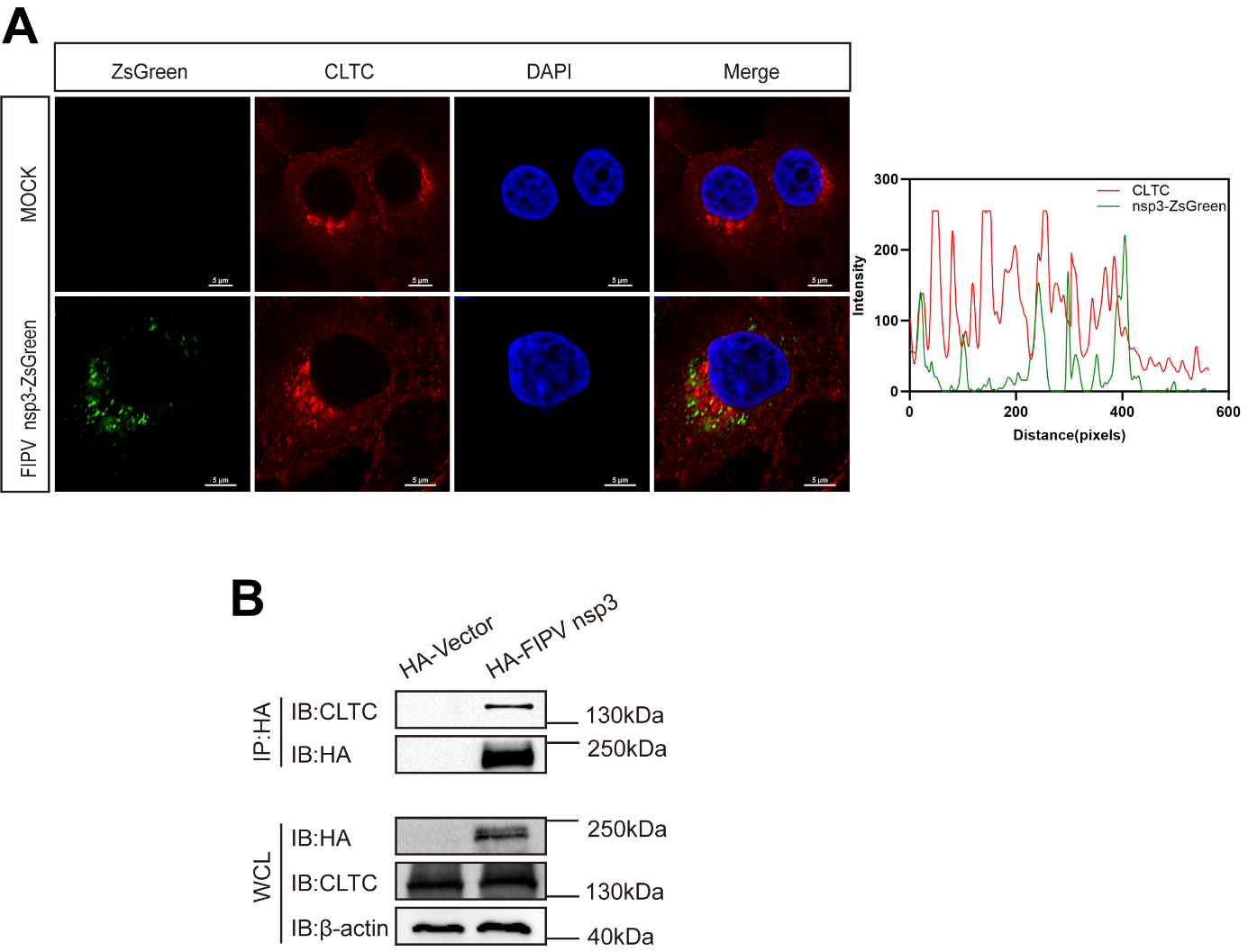


**Figure S3** | Co-localization and interaction between FIPV nsp3 and endogenous CLTC. (A) CRFK cells were mock inoculated or infected with FIPV nsp3-ZsGreen (MOI = 1, 12 hpi), and then fixed. Cells were stanined with anti-CLTC (red) and DAPI (blue). Scale bar = 5 µm. The colocalization of FIPV nsp3-ZsGreen with CLTC was analyzed using Fiji. (B) HEK293T cells were transfected with pCAGGS-HA FIPV nsp3 or empty vector for 48 h. Co-IP was performed using an anti-HA antibody.

**
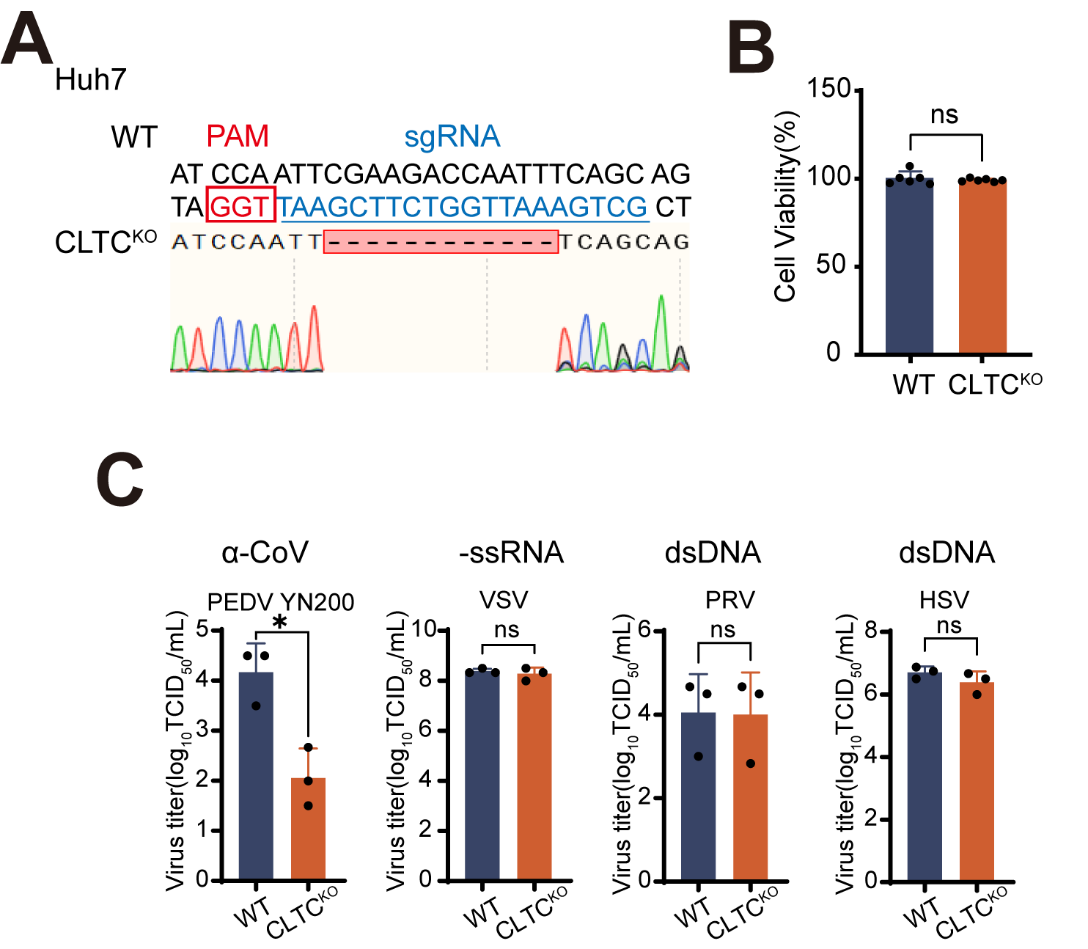
**

**Figure S4** | (A) Sanger sequencing of Huh-7 CLTC knockout cells. (B) Cell viability of Huh-7 WT and CLTC KO cells was determined by MTS assay (n = 6). Data are mean ± SD. (unpaired two-tailed Student’s t-test). ns: p ≥ 0.05. (C) CRFK WT and CLTC KO cells were infected with α-coronavirus PEDV YN200 (MOI = 1), VSV (MOI = 1), PRV (MOI = 1), HSV (MOI = 5) for 12 h. Viral titers in the supernatants were quantified using a TCID_50_ assay (n = 3) on CRFK cells. Data are mean ± SD (unpaired two-tailed Student’s t-test). ns: p ≥ 0.05; *p < 0.05.

**
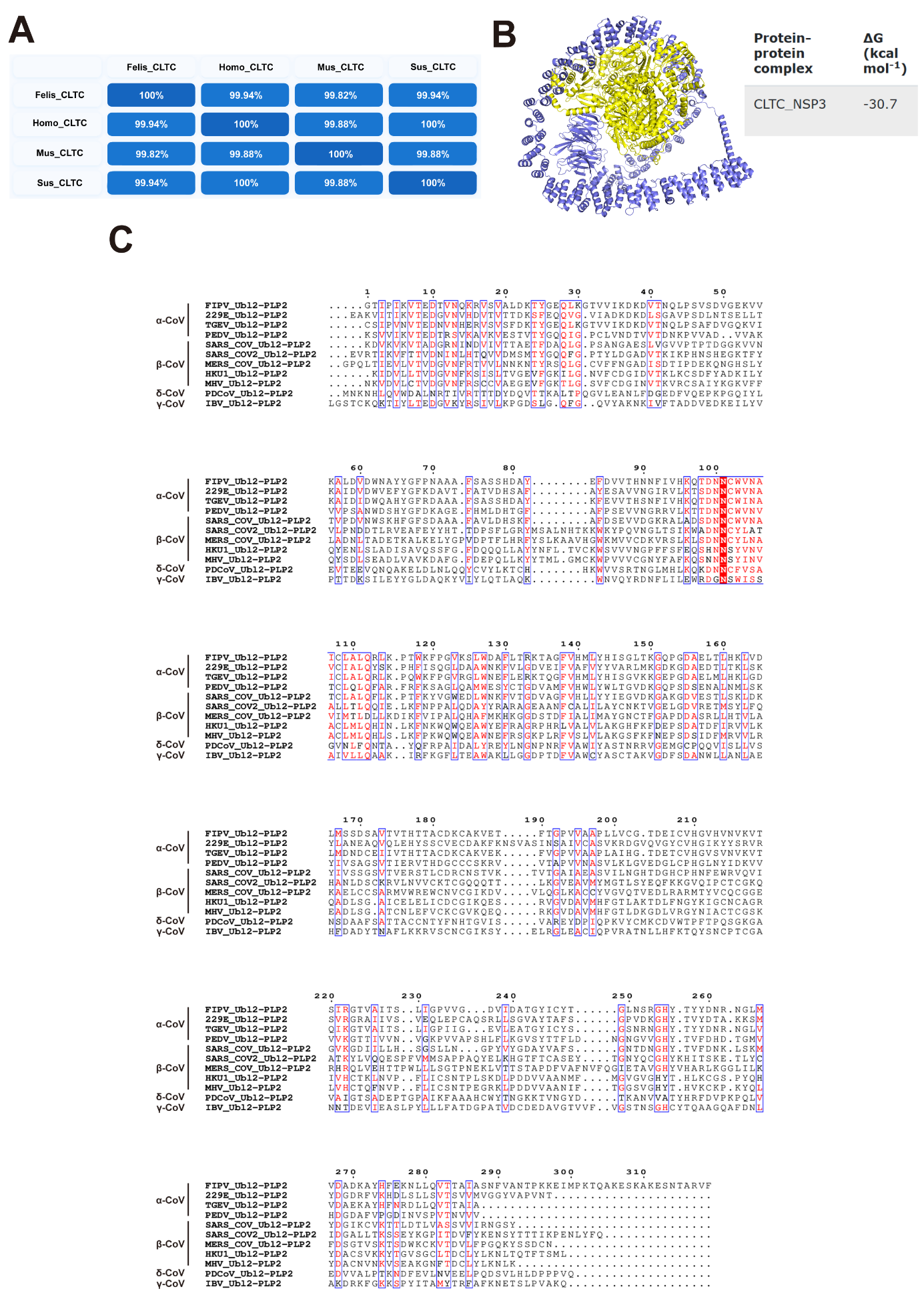
Figure S5** | Homology analysis of CLTC across species and of nsp3 Ubl2-PLP2 across coronaviruses. (A) Homology analysis of CLTC across different species. (B) Molecular docking and structural analysis of nsp3-CLTC complex. Shown is the 3D structural model of the interaction between FIPV nsp3 (yellow) and host CLTC (purple). The structures were predicted by AlphaFold and docked using HADDOCK. The model illustrates a close spatial fit between the viral and host proteins. The calculated binding free energy (ΔG) of the best-scored conformation is -30.7 kcal/mol. (C) Homology analysis of the nsp3 Ubl2-PLP2 domain across diverse coronaviruses.


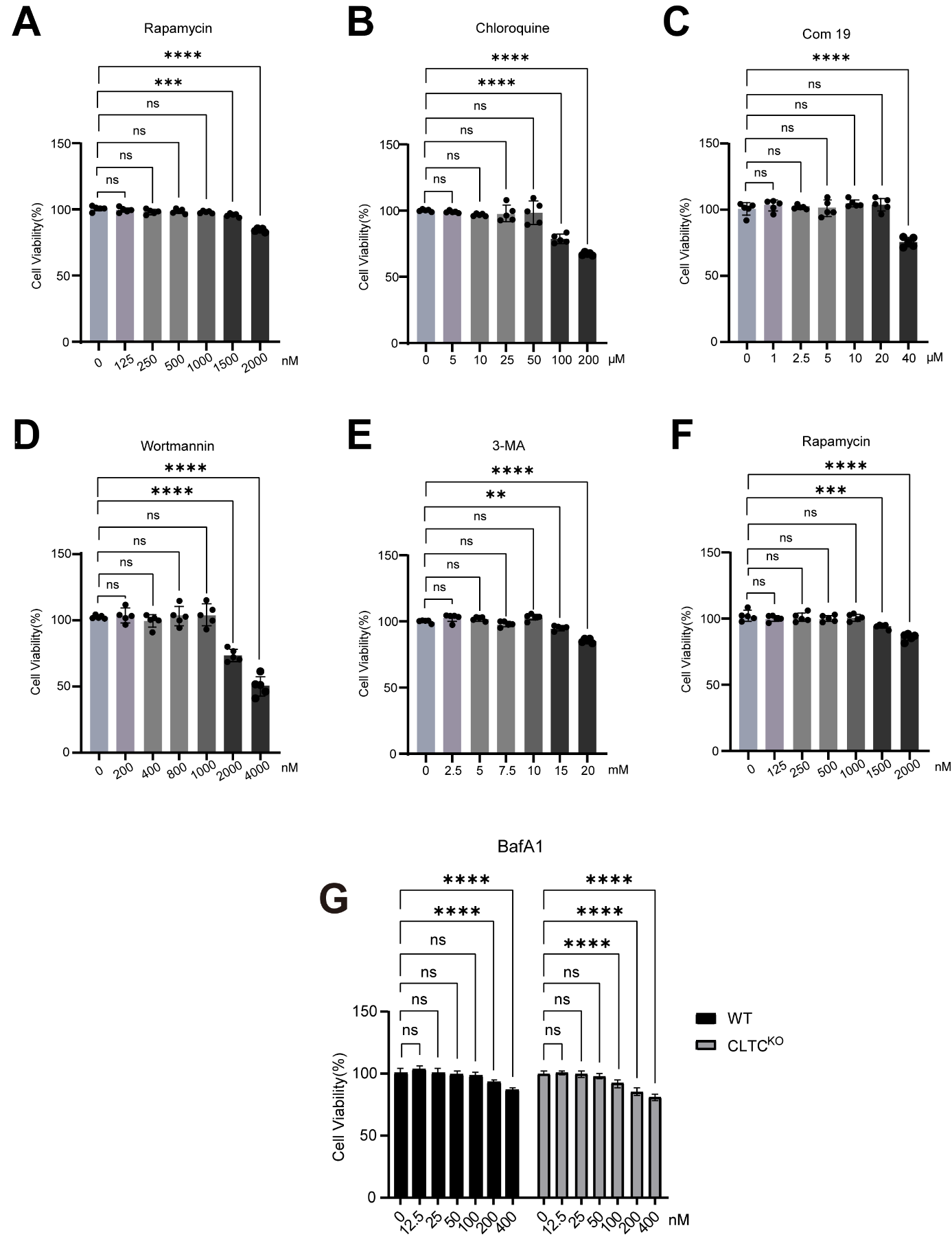


**Figure S6** | Determining cell viability treated with autophagy initiation activators and inhibitors. (A) Cell viability of CLTC KO CRFK cells treated with rapamycin (0, 125, 250, 500, 1000, 1500, 2000 nM) was determined by MTS detection (n = 5). (B-F) Cell viability of WT CRFK cells treated with different concentrations of chloroquine (B), com19 (C), wortmannin (D), 3-MA (E) and rapamycin (F) was determined by MTS detection (n = 5). (G) Cell viability of WT CRFK cells (black) and CLTC KO (grey) cells treated with different concentration of Bafilomycin A1 was determined by MTS detection (n = 6). Data are mean ± SD (one-way ANOVA with Dunnett’s test against the respective control group). ns: p ≥ 0.05; **p < 0.01; ***p < 0.001; ****p < 0.0001.


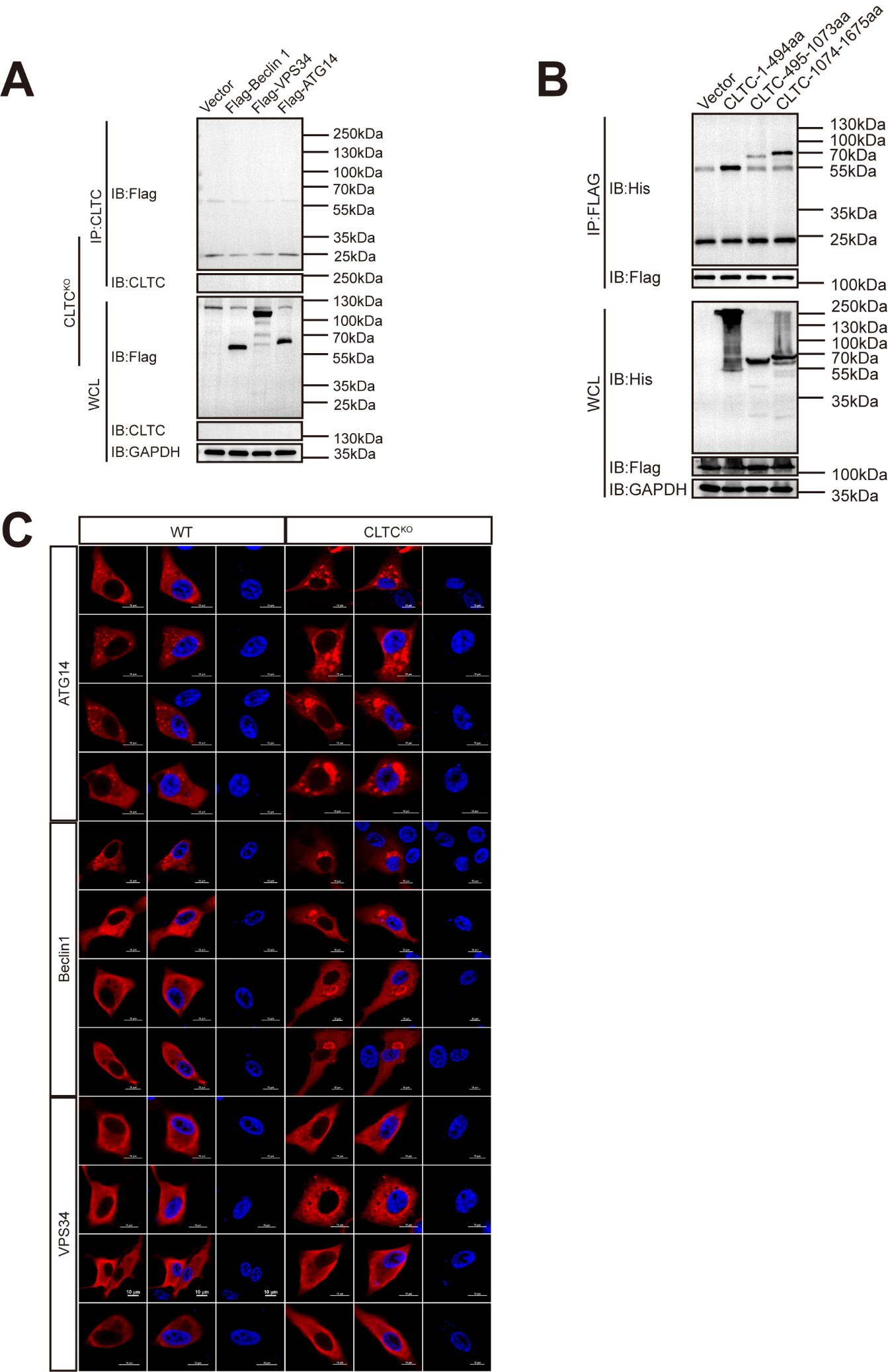


**Figure S7** | Interaction between CLTC and the PI3K complex and their cellular localization. (A) Physical interaction between CLTC and the Class III PI3K complex. HEK293T cells were transfected with Flag-tagged Beclin1, VPS34, or ATG14 for 36 h. In CLTC KO cells, endogenous CLTC was immunoprecipitated (IP: CLTC), and the associated Flag-tagged proteins were detected by immunoblotting (IB: Flag). (B) Mapping the CLTC domains involved in VPS34 interaction. HEK293T cells were co-transfected with Flag-tagged VPS34 and His-tagged CLTC truncation mutants for 36 h. Cell lysates were subjected to immunoprecipitation overnight using anti-Flag magnetic beads, followed by immunoblotting with an anti-His antibody to analyze the interaction profile of CLTC fragments. (C) WT and CLTC KO cells were transfected with Flag-tagged ATG14, Beclin1, or VPS34 (red), and nuclei were stained with DAPI (blue). In WT cells, the proteins exhibit a typical diffuse cytoplasmic distribution.
